# A plant-derived natural photosynthetic system for improving cell anabolism

**DOI:** 10.1038/s41586-022-05499-y

**Published:** 2022-12-07

**Authors:** Pengfei Chen, Xin Liu, Chenhui Gu, Peiyu Zhong, Nan Song, Mobai Li, Zhanqiu Dai, Xiangqian Fang, Zhaoming Liu, Jianfeng Zhang, Ruikang Tang, Shunwu Fan, Xianfeng Lin

**Affiliations:** 1 Department of Orthopaedic Surgery, Sir Run Run Shaw Hospital, Zhejiang University School of Medicine, Hangzhou, China; 2 Key Laboratory of Musculoskeletal System Degeneration and Regeneration Translational Research of Zhejiang Province, Hangzhou, China; 3 Department of Chemistry, Zhejiang University, Hangzhou, China

**Keywords:** Biomaterials - cells, Regenerative medicine

## Abstract

Insufficient intracellular anabolism is a crucial factor involved in many pathological processes in the body^1,2^. The anabolism of intracellular substances requires the consumption of sufficient intracellular energy and the production of reducing equivalents. ATP acts as an ‘energy currency’ for biological processes in cells^3,4^, and the reduced form of NADPH is a key electron donor that provides reducing power for anabolism^5^. Under pathological conditions, it is difficult to correct impaired anabolism and to increase insufficient levels of ATP and NADPH to optimum concentrations^1,4,6–8^. Here we develop an independent and controllable nanosized plant-derived photosynthetic system based on nanothylakoid units (NTUs). To enable cross-species applications, we use a specific mature cell membrane (the chondrocyte membrane (CM)) for camouflage encapsulation. As proof of concept, we demonstrate that these CM-NTUs enter chondrocytes through membrane fusion, avoid lysosome degradation and achieve rapid penetration. Moreover, the CM-NTUs increase intracellular ATP and NADPH levels in situ following exposure to light and improve anabolism in degenerated chondrocytes. They can also systemically correct energy imbalance and restore cellular metabolism to improve cartilage homeostasis and protect against pathological progression of osteoarthritis. Our therapeutic strategy for degenerative diseases is based on a natural photosynthetic system that can controllably enhance cell anabolism by independently providing key energy and metabolic carriers. This study also provides an enhanced understanding of the preparation and application of bioorganisms and composite biomaterials for the treatment of disease.

## Abstract (web summary)

Proof of concept of the viability of a plant-derived photosynthetic system based on nanothylakoid units encapsulated in a chondrocyte membrane to enhance cell anabolism in chondrocytes is demonstrated.

## Main

Intracellular energy and reducing equivalents are deficient under pathological conditions^1,2^. The tricarboxylic acid (TCA) cycle is the major energy metabolic process for ATP generation in most mammalian cells^3,4^. Thus, interventions that target the TCA cycle hold promise to rectify the dysregulated supply of ATP in pathological conditions. However, the TCA cycle involves various metabolic networks, and the delivery of a specific factor that changes its intrinsic pathway may even cause cell death^6^. In addition, the direct provision of exogenous ATP has little effect on cellular metabolism^7^. The reduced form of NADPH can provide reducing power for synthesis reactions and redox balance^5^. Cellular NADPH levels are regulated through the production and utilization of several metabolic pathways (that is, the pentose phosphate pathway, fatty acid oxidation and glutamine metabolism), and direct interventions that target these pathways may lead to cellular metabolic imbalance^1,4^. Moreover, NADPH is expensive, and an uncontrolled supply of NADPH can cause the production of cytotoxic superoxide, which in turn can result in oxidative stress. These properties limit the clinical application of NADPH^8^. Therefore, it is important to construct a controllable and independent ATP and NADPH self-supply system to enhance cell anabolism^9–11^. We propose a systematic top-level design strategy that can be used to treat disease.

Harnessing natural systems for ATP and NADPH production enables new applications. Synthetic liposomes with ATP synthase can establish a proton gradient and drive ATP synthesis^9,12^. Previous studies have also combined thylakoid membranes from spinach and artificial biological networks to realize photosynthetic anabolic reactions on a microscale level^13,14^. However, the use of a controllable and independent natural photosynthetic system to improve cell anabolism has not yet been achieved. Cross-species transplantation of biologically active tissue in vivo also needs to overcome elimination and rejection by the body. In the human body, at the cellular level, various types of immune-related cells (mainly macrophages) are responsible for the clearance of foreign bodies^15^. At the subcellular level (organelles), lysosomes digest and remove foreign bodies by phagocytosis and dissolution^16^. Therefore, avoiding rejection and elimination of a natural photosynthesis system in the mammalian body to achieve a functional cross-species application strategy remains a challenge.

As a key cell structure for mutual recognition and regulation between various cells in the body, the cell membrane plays an essential part in the retention of its inner contents^17^. Vesicles derived from cell membranes can be used to encapsulate specific materials to enhance their biocompatibility and treatment effects^18,19^. Therefore, we considered that the use of a specific mature cell membrane as a camouflage may be an effective strategy to evade cross-species elimination. In turn, in vivo transplantation of an independent natural photosynthetic system can be used to enhance cell anabolism in degenerative diseases.

**Extended Data Fig. 1:**
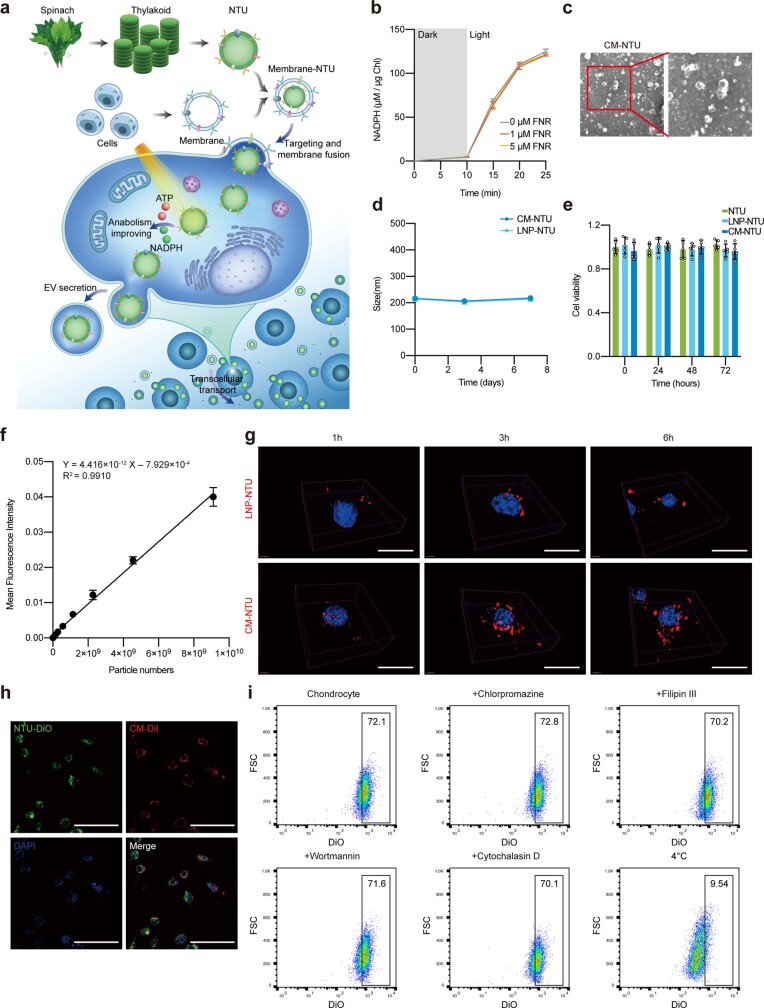
Characterization of CM-NTUs. (a) Schematic diagram of membrane-coated nanothylakoid units (CM-NTUs). (b) NADPH production ability of NTUs *in vitro* with different concentrations of external ferredoxin:NADP+ reductase (FNR) (*n* = 3, mean ± SD). (c) Morphology of CM-NTUs imaged by transmission electron microscopy (TEM) (scale bar, 200 nm). (d) Stability of CM-NTUs and LNP-NTUs over time in phosphate buffered saline (PBS) for 1 week (stored at 4 °C in the dark). (e) Cytotoxicity of NTUs, LNP-NTUs, or CM-NTUs towards chondrocytes (*n* = 5, mean ± SD). (f) Standard curve of DiI fluorescence intensity with respect to NTU particle numbers. Each data point represents the mean of three independent biological replicates. (g) The distribution of LNP-NTUs and CM-NTUs (NTUs labelled with DiI) in chondrocytes at 1, 3, and 6 h observed by structural illumination microscopy (SIM) (scale bar, 10 μm; nuclei, blue; NTUs, red). (h) Fluorescent images of CM-NTU (CM labelled with DiI and NTUs labelled with DiO) uptake by chondrocytes for 1 hour (scale bar, 30 μm; nuclei, blue; NTUs, green; CM, red). (i) DiO-positive chondrocytes assessed by flow cytometry after treatment with CM-NTUs (DiO-labelled NTUs) for 6 h. *n* represents the number of biologically independent samples. Source data

Osteoarthritis is a common degenerative disease, and pathological chondrocytes exhibit ATP and NADPH depletion^20^ and increased production of reactive oxygen species (ROS) and matrix metalloproteinases (MMPs)^21^. Chondrocytes with an energy deficit exhibit decreased synthesis of extracellular matrix (ECM) proteins, including collagen and proteoglycans^22,23^. The loss of energy reserves within chondrocytes coupled with a shift in metabolic pathways towards glycolysis contributes to impaired ECM synthesis and anabolism in pathological chondrocytes^24^. Current treatments cannot systematically correct the metabolic imbalance that occurs in degenerated chondrocytes and are associated with poor clinical outcomes^25^. This study utilizes a commonly used mouse model of osteoarthritis to conduct proof-of-concept research. We nanoencapsulated NTUs with a chondrocyte-derived membrane to produce CM-NTUs. We aim to avoid elimination in the body and to improve cell anabolism of degenerated cartilage to treat osteoarthritis (Extended Data Fig. 1a).

## Preparation and characterization of CM-NTUs

**Fig. 1:**
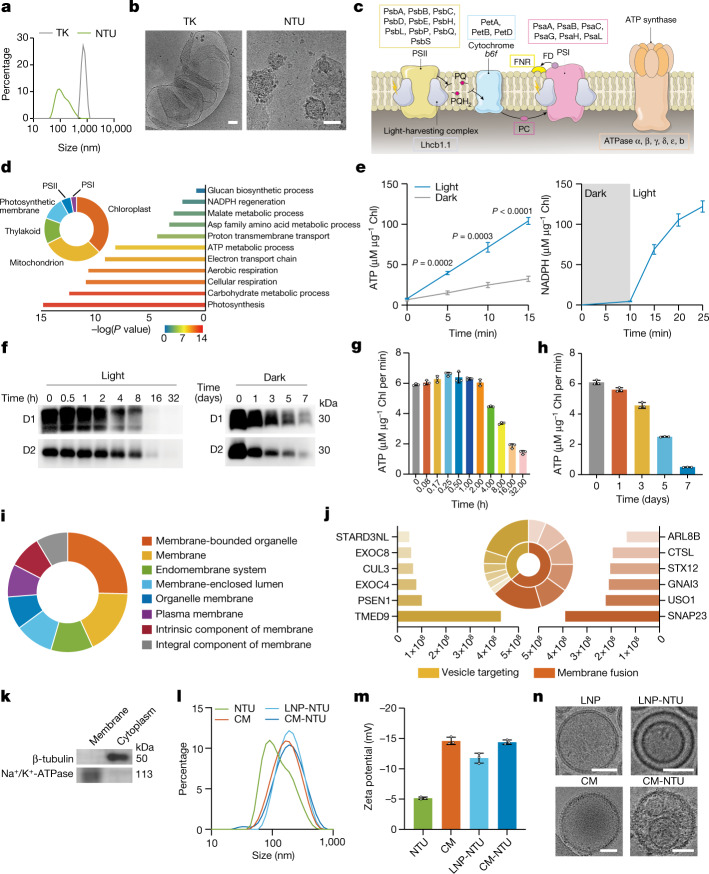
Preparation and characterization of CM-NTUs. **a**, Diameters of thylakoid (TK) organelles and NTUs. **b**, Cryo-TEM images of thylakoid organelles and NTUs. **c**, Schematic illustration of photosynthesis light reaction-associated proteins and the photosynthetic electron transport chain in NTUs. FD, ferredoxin; PC, plastocyanin; PSI, photosystem I; PSII, photosystem II; PQ, plastoquinone. **d**, Proteomics analysis of NTUs. The identified proteins were classified according to their cellular components and biological processes and analysed using protein analysis through evolutionary relationships (PANTHER) overrepresentation test with Fisher’s exact test for significance. **e**, ATP and NADPH production capacity of NTUs in vitro (*n* = 3, mean ± s.d.). **f**, Immunodetection of D1 and D2 abundance in NTUs under light illumination for 0–32 h (80 µmol photons m^−2^ s^−1^) or darkness for 0–7 days (at room temperature). Uncropped gel is in Supplementary Fig. 1a. Similar results were obtained from three biologically independent samples. **g**,**h**, ATP production of NTUs was measured under light illumination (**g**) for 0–32 h (80 µmol photons m^−2^ s^−1^) or in the dark (**h**) for 0–7 days (at room temperature) (*n* = 3, mean ± s.d.). **i**, Proteomics analysis of CM. The identified proteins were classified according to their cellular components. **j**, Content and categories of proteins in the CM involved in vesicle targeting and membrane fusion. **k**, Western blot analysis of Na^+^/K^+^-ATPase and β-tubulin in CM and cytoplasm. Na^+^/K^+^-ATPase was significantly enriched, and β-tubulin was present at low levels on the CM. Uncropped gel is in Supplementary Fig. 1b. **l**, Diameters of NTUs, CM, LNP-NTUs and CM-NTUs. **m**, Zeta potential of NTUs, CM, LNP-NTUs and CM-NTUs (*n* = 3, mean ± s.d.). **n**, Cryo-TEM images of LNPs, LNP-NTUs, CM and CM-NTUs. *n* represents the number of biologically independent samples. *P* values are indicated on the graph and were determined using two-tailed *t*-test (**e**). Scale bars, 50 nm (**n**) or 100 nm (**b**). Source data

The obtained NTUs were first analysed. The particle size of the thylakoid membrane obtained was about 758 nm, and the particle size of the NTUs was around 130 nm (Fig. 1a). The results of cryogenic transmission electron microscopy (cryo-TEM) analyses further confirmed the nanostructure of the NTUs (Fig. 1b). Proteomics results showed that the NTUs retained all the protein components required for photosynthesis on the surface of the thylakoid membrane (Fig. 1c and Supplementary Table 1). NTU-specific peptides corresponding to photosynthetic activity-related proteins were also observed (Supplementary Table 2). Gene Ontology (GO) cellular component analysis indicated that 77 proteins were closely related to photosynthetic membranes and 87 proteins belonged to thylakoids (Fig. 1d). The GO biological process analysis suggested that the NTU components are involved in NADPH regeneration and ATP metabolic processes (Fig. 1d). NTUs were able to catalyse the production of ATP from ADP after exposure to light. In detail, they had a specific activity of 6.4 ± 0.1 µM min^−1^ μg^−1^ total chlorophyll (Chl, sum of Chl A and B; mean ± s.d.) (approximately 2.7 × 10^−11^ μM min^−1^ per NTU), which was significantly faster than production under dark conditions (1.7 ± 0.4 µM min^−1^ μg^−1^ Chl; mean ± s.d.) (Fig. 1e). Moreover, the NTUs were able to catalyse the light-dependent reduction of NADP^+^ to NADPH, with a specific activity of 7.8 ± 0.4 µM min^−1^ μg^−1^ Chl (mean ± s.d.; about 3.3 × 10^−11^ μM min^−1^ per NTU) (Fig. 1e). The addition of external ferredoxin:NADP^+^ reductase (FNR) did not increase the rate of NADPH production by NTUs (Extended Data Fig. 1b). This is potentially because FNR is anchored to the thylakoid membrane and the membrane-bound FNR was sufficient to generate NADPH in the system^26,27^. We measured the abundance of D1 and D2 proteins in isolated NTUs over time. Under light illumination, both D1 and D2 proteins were completely degraded within 8–16 h (Fig. 1f). Under dark conditions, both proteins were almost completely degraded within 5–7 days (Fig. 1f). We then measured changes in ATP production capacity of the NTUs over time. The capacity of NTUs to produce ATP decreased significantly after 16 h of light exposure or after 7 days of storage in the dark (Fig. 1g,h). Overall, the change in the capacity of NTUs to produce ATP under light and dark conditions was consistent with the change in protein degradation levels.

We next purified and obtained CM samples and subjected them to proteomics analysis (Supplementary Table 3). The GO cellular component analysis indicated that the protein components were mainly related to the structure of the cell membrane (Fig. 1i). The potential functions were significantly correlated with vesicle targeting (that is, cullin-3 and exocyst complex component 4) and membrane fusion (that is, general vesicular transport factor p115 and guanine nucleotide-binding protein G_i_subunit alpha-3) (Fig. 1j). The results of western blot analyses further verified the successful isolation of the CM (Fig. 1k). Next, we physically extruded the purified CM and the NTUs together to obtain CM-coated NTUs. Both the size (around 216 nm) and surface zeta potential data showed that the NTUs were successfully coated by the cell membrane (Fig. 1l,m). The core–shell structure of the CM-NTUs was confirmed by visualizing samples under cryo-TEM and transmission electron microscopy (TEM) (Fig. 1n and Extended Data Fig. 1c). We also used lipid nanoparticles (LNPs) to encapsulate the NTUs (LNP-NTUs) as a non-cell membrane control group (Fig. 1l–n).

## Assessment of the cellular uptake of CM-NTUs

**Fig. 2:**
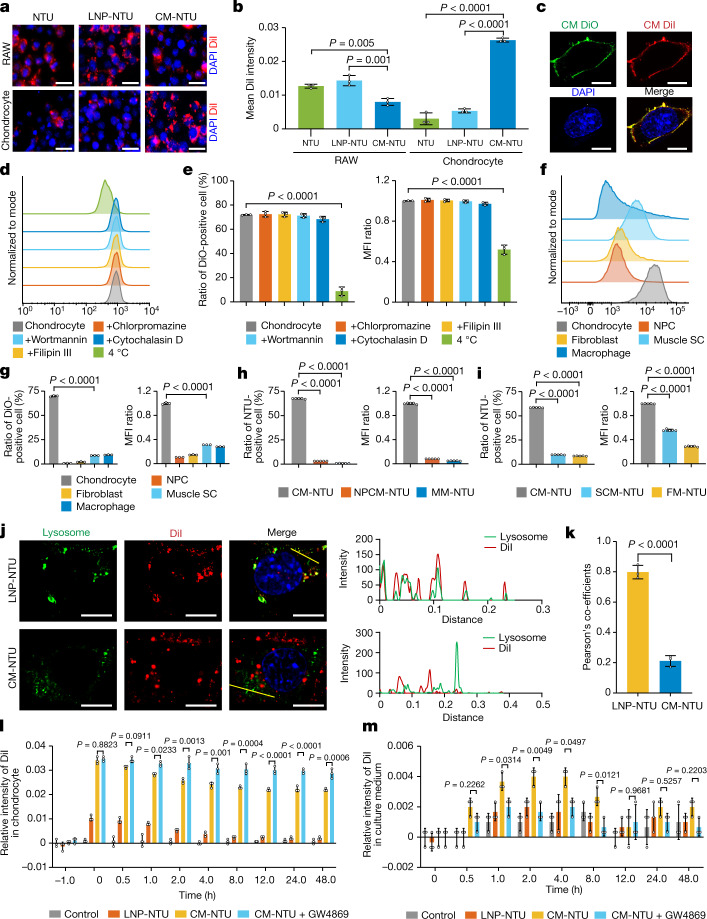
Cell membrane fusion and intracellular release process of CM-NTUs. **a**, Uptake of NTUs, LNP-NTUs and CM-NTUs (NTUs labelled with DiI) by RAW 264.7 macrophages (RAW) or chondrocytes. Nuclei, blue; NTUs, red. **b**, The fluorescence intensity of NTU, LNP-NTU and CM-NTU uptake by RAW 264.7 macrophages or chondrocytes (*n* = 3, mean ± s.d.). **c**, Fluorescent images indicating the interaction between DiO-labelled CM and DiI-labelled CM. Nuclei, blue; DiO-labelled CM, green; Dil-labelled CM, red. **d**, DiO-positive chondrocytes measured by flow cytometry after treatment with CM-NTUs (DiO-labelled NTUs). Chondrocytes were cooled to 4 °C or separately pretreated with endocytosis-related inhibitors at 37 °C. **e**, Ratio of DiO-positive chondrocytes (left) and mean fluorescence intensity (MFI) (right) of DiO (*n* = 3, mean ± s.d.). **f**, Flow cytometry analysis of five types of cultured cells after incubation with CM-NTUs (DiO-labelled NTUs). **g**, Ratio of DiO-positive cells (left) and MFI (right) of DiO in five cell types (*n* = 3, mean ± s.d.). **h**,**i**, Ratio of chondrocytes taking up five different coated NTUs and the corresponding MFI values in staining scheme 1 (**h**) and scheme 2 (**i**) (*n* = 5, mean ± s.d.). FM-NTU, NTUs coated with fibroblast membrane; MM-NTU, NTUs coated with macrophage membrane. **j**, Fluorescent visualization (left) of NTU localization in chondrocytes 6 h after incubation with LNP-NTUs or CM-NTUs (NTUs, red; nuclei, blue; lysosome, green) and intensity profiles (right) across the cell along the selected line (yellow line). **k**, Pearson’s correlation coefficients of the NTUs and lysosomes (*n* = 3, mean ± s.d.). **l**,**m**, Quantitative detection of DiI fluorescence intensity in chondrocytes (**l**) and culture medium (**m**) (*n* = 3, mean ± s.d.). *n* represents the number of biologically independent samples. *P* values are shown in graphs and were determined using one-way analysis of variance (ANOVA) (**b**,**e**,**g**–**i**,**l**,**m**) or two-tailed *t*-test (**k**). Scale bars, 3 μm (**c**,**j**), 20 μm (**a**). Source data

The long-term stability of CM-NTUs and LNP-NTUs was evaluated, and both nanoparticles showed small changes in size over 1 week (Extended Data Fig. 1d). Before starting the cell and animal experiments, we incubated chondrocytes with the NTUs, LNP-NTUs and CM-NTUs, and the concentrations of these particles had no impact on the health of the chondrocytes (Extended Data Fig. 1e). To estimate the average number of NTUs in cells, we obtained a standard curve of fluorescence intensity (labelling NTUs with 1,1′-dioctadecyl-3,3,3,3′-tetramethylindocarbocyanine perchlorate (DiI)) and the corresponding number of NTUs (Extended Data Fig. 1f). We then incubated mouse macrophages or chondrocytes with NTUs, LNP-NTUs or CM-NTUs. The intensity of fluorescence originating from NTUs or LNP-NTUs in macrophages was greater than that of CM-NTUs. This result suggests that the CM coating significantly reduced the interaction between the NTUs and macrophages (Fig. 2a,b). CM-NTUs were more efficiently internalized by chondrocytes compared with NTUs or LNP-NTUs (Fig. 2a,b). We observed higher internalization of CM-NTUs in chondrocytes compared with LNP-NTUs (Extended Data Fig. 1g). These results indicate the selective targeting ability of the CM-NTUs. Combined with the proteomics analysis results of the vesicle targeting-related proteins found in CMs, these proteins may also enhance the targeting process of CM-NTUs.

**Extended Data Fig. 2:**
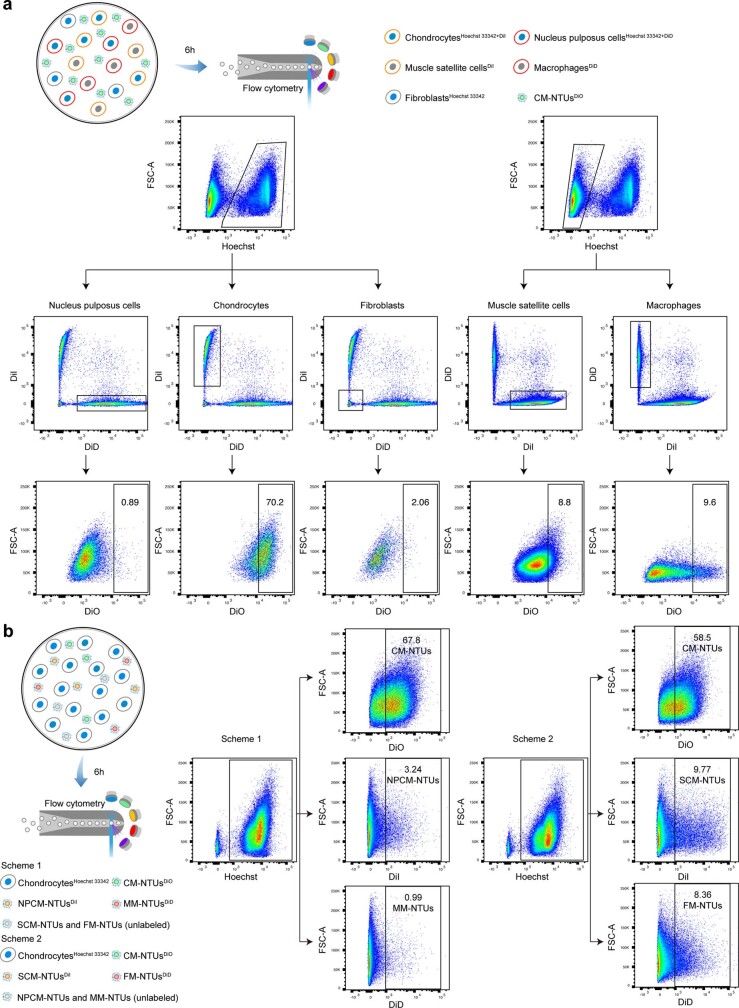
The quantitative selectivity with which cells take up NTUs. (a) Gating strategies of five different cell types. Hoechst 33342^+^ DiI^-^ DiD^+^ cells were defined as nucleus pulposus cells (NPCs). Hoechst 33342^+^ DiI^+^ DiD^-^ cells were defined as chondrocytes. Hoechst 33342^+^ DiI^-^ DiD^-^ cells were defined as fibroblasts. Hoechst 33342^-^ DiI^+^ DiD^-^ cells were defined as muscle satellite cells (SCs). Hoechst 33342^-^ DiI^-^ DiD^+^ cells were defined as macrophages. The following results indicate the proportions of the five types of cells that took up CM-NTUs: 69.9% (chondrocytes), 0.9% (NPCs), 2.2% (fibroblasts), 8.8% (SCs), and 9.4% (macrophages). Based on the level of mean fluorescence intensity (MFI) and using 5.8 × 10^4^ NTUs per chondrocyte as the standard, we estimated that the numbers of NTUs taken up by NPCs, fibroblasts, SCs, and macrophages were ~6.4 × 10^3^, 8.6 × 10^3^, 1.8 × 10^4^, and 1.6 × 10^4^ per cell, respectively. (b) Gating strategies of chondrocytes. Scheme #1 (left panel): Hoechst 33342^+^ DiO^+^ cells were defined as chondrocytes taking up CM-NTUs. Hoechst 33342^+^ DiI^+^ cells were defined as chondrocytes taking up NPC membrane-NTUs (NPCM-NTUs). Hoechst 33342^+^ DiD^+^ cells were defined as chondrocytes taking up macrophage membrane-NTUs (MM-NTUs). Scheme #2 (right panel): Hoechst 33342^+^ DiO^+^ cells were defined as chondrocytes taking up CM-NTUs. Hoechst 33342^+^ DiI^+^ cells were defined as chondrocytes taking up SC membrane-NTUs (SCM-NTUs). Hoechst 33342^+^ DiD^+^ cells were defined as chondrocytes taking up fibroblast membrane-NTUs (FM-NTUs). The results showed that chondrocytes took up the highest percentage of CM-NTUs, at 67.6% and 58.7% in the two experiments, respectively. The proportions of chondrocytes that took up the NPCM-NTUs, MM-NTUs, SCM-NTUs, and FM-NTUs were 3.2%, 1.0%, 9.9%, and 8.6%, respectively. Based on the MFI, we estimated the number of NPCM-NTUs, MM-NTUs, SCM-NTUs, and FM-NTUs taken up per chondrocyte was ~4.8 × 10^3^, 2.9 × 10^3^, 3.2 × 10^4^, and 1.7 × 10^4^, respectively.

We then studied the intracellular trafficking properties of the CM-NTUs. The CM mainly accumulated in the outer cellular layer, whereas the NTUs were concentrated in the interior (Extended Data Fig. 1h). Moreover, the results showed that the CM merged with homologous chondrocytes (Fig. 2c). Therefore, we proposed that the integration pathway of the CM-NTUs might be similar to that of an enveloped virus, which initiates cellular internalization through membrane fusion and then releases the enclosed capsid into the cytosol^28,29^. Flow cytometry analysis showed that although low temperature suppressed the cellular uptake of CM-NTUs, the addition of endocytosis-related inhibitors did not have an affect (Fig. 2d,e and Extended Data Fig. 1i). These findings indicate that the CM-NTUs might enter chondrocytes predominantly through a membrane fusion mechanism. Combined with the results from the proteomics analysis of CM, the identified membrane-fusion-related proteins may promote the fusion process. We then investigated the selectivity with which cells take up NTUs coated with their own cell-type membranes. In the first experimental model, we added CM-NTUs to cultures of five different cell types (nucleus pulposus cells (NPCs), chondrocytes, fibroblasts, muscle satellite cells (SCs) and macrophages) (Extended Data Fig. 2a). The results indicated that chondrocytes specifically take up CM-NTUs compared with the other cells (Fig. 2f,g). We then coated NTUs with membranes from various cell types and incubated them with chondrocytes (Extended Data Fig. 2b). The results showed that the chondrocytes took up CM-NTUs to the highest percentage compared with NTUs coated with other membranes (Fig. 2h,i).

Most foreign bodies internalized by cellular endocytosis are further trafficked into lysosomes, in which they are then degraded^30^. In this study, only a few signals of internalized CM-NTUs were colocalized with lysosomes (Fig. 2j,k). By contrast, most of the internalized LNP-NTUs colocalized with lysosomes (Fig. 2j,k). These results suggest that the CM-NTUs bypass the endocytic pathway and avoid lysosomal degradation.

**Extended Data Fig. 3:**
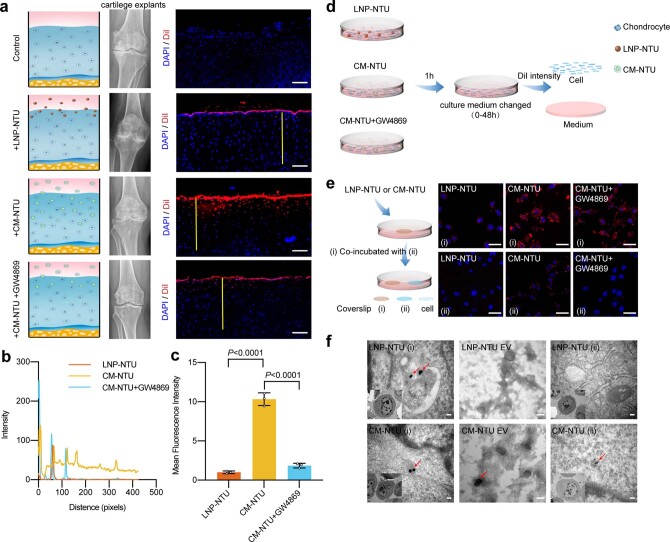
Superior tissue penetration of the CM-NTUs. (a) Degenerated tissues and organs often exhibit fibrosis and densification of the ECM. To deliver an intra-articular injected nanomedicine to the cytosol of chondrocytes in knee joints, researchers must overcome the biological barrier for deep penetration into the avascular and dense degenerated cartilage^58^. Cartilage explants from OA patients were examined to assess the depth of CM-NTU or LNP-NTU (NTUs labelled with DiI) penetration after 24 h. For the inhibitor group, cartilage explants were pretreated with an extracellular vesicle (EV) secretion inhibitor for 24 h before CM-NTU incubation (scale bar, 100 μm; nuclei, blue; NTUs, red). (b) DiI fluorescence intensity profiles across the section along the selected line (indicated by a yellow line in the inset image of a). (c) Mean fluorescence intensity of cartilage sections (*n* = 3, mean ± SD). (d) Schematic diagram of the detection of DiI fluorescence intensity in chondrocytes and culture medium. (e) Intracellular transfer of CM-NTUs or LNP-NTUs (NTUs labelled with DiI) visualized by confocal microscopy (scale bar, 20 μm; NTUs, red; nuclei, blue). Chondrocytes on coverslips (i) were cultured in medium that contained CM-NTUs or LNP-NTUs for 6 h. A coverslip (i) was rinsed and imaged. Then, fresh culture medium was added along with a coverslip with fresh cells on the coverslip (ii) for 24 h. For the inhibitor group, chondrocytes were pretreated with an EV secretion inhibitor for 24 h. The DiI signal from the CM-NTUs was high in the cells on coverslips (ii), indicating that some of the NTUs taken up in the cells on coverslips (i) were transported into the medium and subsequently internalized by the cells on coverslips (ii). In contrast, the NTUs from the LNP-NTU-treated cells exhibited limited transportation to the cells on coverslips (ii). Moreover, pretreatment with GW4869 blocked the transcellular transmission effect of the CM-NTUs. (f) Intracellular transfer of CM-NTUs or LNP-NTUs (gold nanoparticles encapsulated into NTUs) visualized by transmission electron microscopy (TEM) (scale bar, 200 nm). The processing of chondrocytes on the coverslip is the same as that described in e. The EVs secreted by the chondrocytes on the coverslip (i) were collected separately for TEM observation. The cells stimulated by the CM-NTUs could secrete EVs containing gold nanoparticles, and gold nanoparticles could be observed in the cells on coverslips (ii). In contrast, no gold nanoparticles were observed in EVs secreted by the LNP-NTU-stimulated cells or the cells on coverslips (ii). *n* represents the number of human specimens. *P* values are indicated in the graph and were determined using one-way ANOVA (c). Source data

We used cartilage explants from patients with osteoarthritis to evaluate the penetration of CM-NTUs and LNP-NTUs. After 24 h of exposure, the CM-NTUs were evenly distributed throughout the cartilage explant, whereas the LNP-NTUs were restricted to its surface (Extended Data Fig. 3a,b). Moreover, the CM-NTUs exhibited a higher fluorescence intensity than the LNP-NTUs (Extended Data Fig. 3c). These results indicated that the CM-NTUs are able to penetrate degenerated cartilage compared with LNP-NTUs. Studies have reported that nanoparticles can achieve rapid penetration through transcellular transport^31,32^. Furthermore, cells can communicate with neighbouring cells through the secretion of extracellular vesicles (EVs)^33^. Here we found that after pretreatment with GW4869, both the penetration depth and fluorescence intensity of the CM-NTUs were significantly decreased (Extended Data Fig. 3a–c). Next, we cultured chondrocytes with CM-NTUs or LNP-NTUs (Extended Data Fig. 3d). The fluorescence intensity in cells was increased in the CM-NTU group compared with the LNP-NTU group and the control group (Fig. 2l). However, pretreatment with GW4869 slowed the decrease in fluorescence intensity in chondrocytes (Fig. 2l). Notably, the culture medium exhibited an increase in fluorescence signal over time, and the CM-NTU group exhibited a significantly increased fluorescence signal than the CM-NTU plus GW4869 group during the first 8 h of culture (Fig. 2m). The EV-dependent transcellular transport of the CM-NTUs was further confirmed by ‘infection’ between different batches of cells (Extended Data Fig. 3e,f). On the basis of these results, we propose that after entering chondrocytes, a portion of the NTUs may be secreted out again by cells in the form of EVs, particularly at the early stage, which may be essential for subsequent uptake and secretion for active penetration.

## CM-NTUs improve cell anabolism

**Fig. 3:**
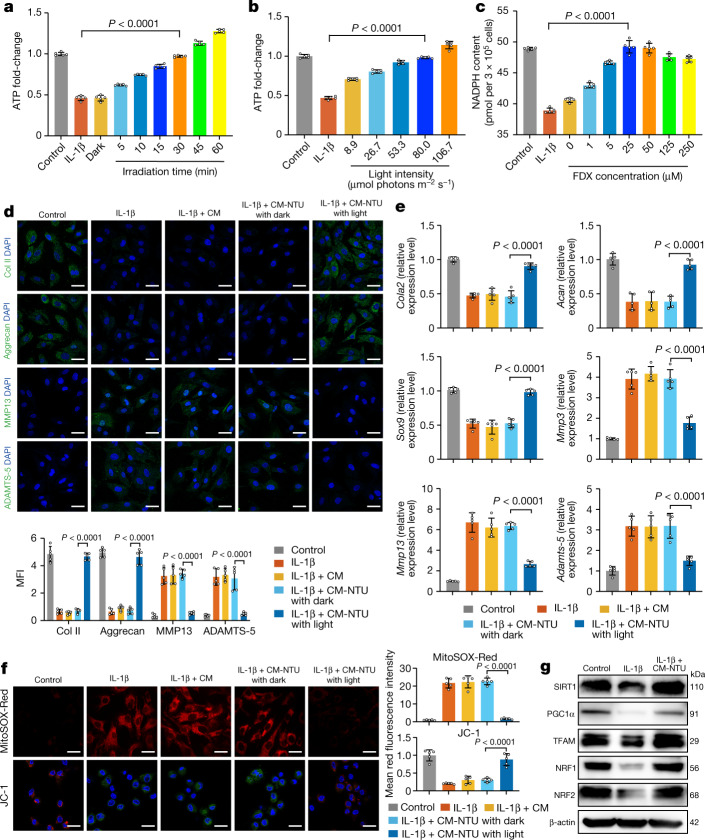
CM-NTUs improve cell anabolism. **a**, ATP levels of chondrocytes treated with CM-NTUs and red light irradiation (80 µmol photons m^−2^ s^−1^) for different time intervals (*n* = 5, mean ± s.d.). **b**, ATP levels of chondrocytes treated with CM-NTUs and red light irradiation for 30 min under different light intensities (*n* = 5, mean ± s.d.). **c**, NADPH levels of chondrocytes treated with CM-NTUs with different encapsulated ferredoxin (FDX) concentrations (*n* = 5, mean ± s.d.). **d**, Immunofluorescence staining (top) and quantification (bottom) of Col II, aggrecan, MMP13 and ADAMTS-5 levels in chondrocytes (*n* = 5, mean ± s.d.). Mouse chondrocytes were stimulated with IL-1β for 24 h followed by CM or CM-NTU treatment for 6 h with or without red light irradiation (80 µmol photons m^−2^ s^−1^, 30 min). **e**, PCR with reverse transcription detection of *Col2a1*, *Acan*, *Sox9*, *Mmp3*, *Mmp13* and *Adamts5* expression in chondrocytes incubated with IL-1β, IL-1β and CM, or IL-1β and CM-NTUs in the dark or with IL-1β and CM-NTUs in the light (*n* = 5, mean ± s.d.). **f**, MitoSOX-Red and JC-1 staining (left) and quantification (right) of chondrocytes incubated with IL-1β, IL-1β and CM, or IL-1β and CM-NTUs in the dark or with IL-1β and CM-NTUs in the light (MitoSOX-Red, red; JC-1, red and green; *n* = 5, mean ± s.d.). **g**, Western blots of the mitochondrial biogenesis markers SIRT1, PGC1α, TFAM, NRF1 and NRF2. Chondrocytes were incubated with IL-1β or with IL-1β and CM-NTUs in the light. Uncropped gel is in Supplementary Fig. 1d. *n* represents the number of biologically independent samples. *P* values are indicated in graphs and were determined using one-way ANOVA (**a**–**f**). Scale bars, 10 μm (**d**,**f**). Source data

Given the successful fabrication of CM-NTUs, the light-controllable effect of these on cell anabolism was assessed. We chose interleukin-1β (IL-1β) to induce metabolic impairment in mouse chondrocytes^34^. We first incubated the IL-1β-treated chondrocytes with the CM-NTUs under different light conditions and then tracked cellular ATP and NADPH changes over time. We adjusted the light intensity, the irradiation time of the light and the encapsulated ferredoxin concentration in the CM-NTUs to optimize the experimental conditions. CM-NTUs exposed to red light (80 µmol photons m^−2^ s^−1^) irradiation for 30 min and with 25 μM encapsulated ferredoxin (diluted to about 1.2 μM after delivery into cells) were selected for subsequent experiments. Under these conditions, CM-NTUs restored intracellular ATP and NADPH levels close to those noted in control chondrocytes (Fig. 3a–c).

**Extended Data Fig. 4:**
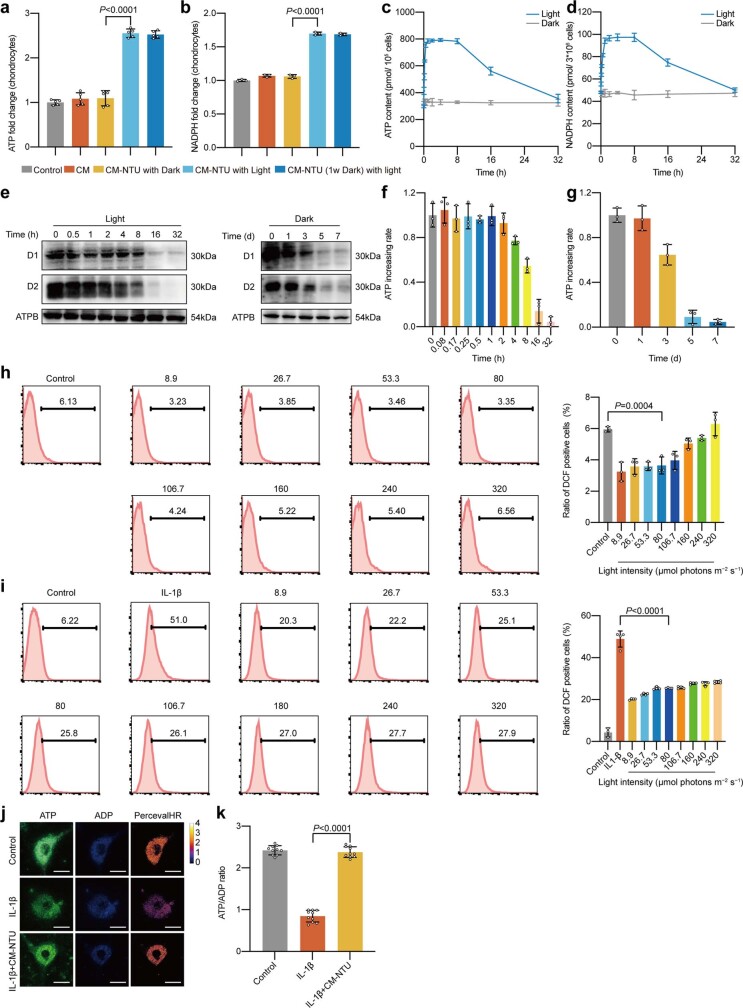
CM-NTUs regulate the metabolic homeostasis of chondrocytes. (a, b) ATP (a) and NADPH (b) levels in mouse chondrocytes incubated with CM, CM-NTUs in the dark, CM-NTUs in the light, or CM-NTUs (stored at 4 °C for 1 week) in the light (*n* = 5 for ATP detection, *n* = 3 for NADPH detection, mean ± SD). The CM-NTUs with light exposure increased the intracellular ATP and NADPH levels of chondrocytes by 2.6- and 1.7-fold, respectively. Storage for 1 week did not affect the ability of the CM-NTUs to enhance intracellular ATP and NADPH production. (c,d) Changes in the levels of ATP (c) and NADPH (d) over time in illuminated and unilluminated cells incubated with CM-NTUs (*n* = 3, mean ± SD). In unilluminated cells, CM-NTUs do not alter cellular ATP levels in contrast to that noted in the cell-free system (where a small amount of ATP can be produced in the absence of light due to the membrane-bound adenylate kinase of thylakoids^13^). This finding might be attributed to the multiple adenylate kinase isoenzymes (adenylate kinase 1–9) in mammalian cells^59,60^ and because the intracellular reaction had already reached equilibrium before the addition of CM-NTUs. (e) Immunodetection of D1 and D2 abundance in chondrocytes treated with NTUs under light illumination for 0-32 h (at 37 °C, 80 µmol photons m^−2^ s^−1^) or in the dark for 0–7 days (at 37 °C). Both the D1 and D2 proteins in chondrocytes were completely degraded in 8–16 h under light illumination. Under dark conditions, both protein levels decreased gradually from 0 to 3 days and were almost undetectable at 5 days. For gel source data, see Supplementary Fig. 1c. Similar results were obtained from three biologically independent samples. (f,g) Stability of intracellular NTUs in light or dark conditions: Changes in the ability of NTUs to increase intracellular ATP under light illumination (f) for 0–32 h (at 37 °C, 80 µmol photons m^−2^ s^−1^) or in the dark (g) for 0-7 days (at 37 °C) (*n* = 3, mean ± SD). In the relatively stable period, NTUs taken up by chondrocytes were stable for at least 2 h under continuous light illumination (with an ATP increase rate of 17.7 ± 1.4 pmol min^−1^ per 10^5^ cells at 2 h, ~93% compared to 0 h). In contrast, NTUs in chondrocytes kept in the dark for 24 h were stable. Upon restimulation with light, the ATP increase rate reached 17.5 ± 1.6 pmol min^−1^ per 10^5^ cells (~97% compared to 0 days). After 2 h of light or 24 h of dark, the ability of NTUs to increase ATP entered the rapid decline period. After continuous light stimulation for 16 h or storage in the dark for 5 days, the NTUs almost completely lost their photosynthetic capacity to increase the ATP concentration in the cells. (h) Effect of CM-NTUs on the ROS levels of chondrocytes after light illumination with different intensities (8.9–320 µmol photons m^−2^ s^−1^) (*n* = 3, mean ± SD). The ratio of DCF-positive cells decreased from 5.9% (in normal chondrocytes) to 3.6% (in normal chondrocytes containing NTUs) under a light intensity of 80 µmol photons m^−2^ s^−1^. Even under a light intensity of 320 µmol photons m^−2^ s^−1^, the ratio of DCF-positive cells (6.3%) was not significantly increased compared with the baseline value. (i) Effect of CM-NTUs on the ROS levels of chondrocytes incubated with IL-1β. Chondrocytes containing NTUs were illuminated with different intensities (8.9–320 µmol photons m^−2^ s^−1^) (*n* = 3 for control, *n* = 4 for other samples, mean ± SD). In degenerative chondrocytes induced by IL-1β treatment, the ratio of DCF-positive cells increased to 48.9%. After the degenerative chondrocytes containing NTUs were treated with light illumination (80 µmol photons m^−2^ s^−1^), the ratio of DCF-positive cells decreased to 25.6%. Under a higher light intensity (320 µmol photons m^−2^ s^−1^), the ratio of DCF-positive cells (28.4%) remained significantly lower than that of IL-1β-treated cells. (j, k) Fluorescence image (j) and quantitative analysis (k) of the ATP/ADP ratio of cells after different treatments (scale bar, 10 μm; *n* = 10, mean ± SD). Chondrocytes were incubated with IL-1β or IL-1β + CM-NTUs in the light. The decrease in the cytoplasmic ATP/ADP ratio caused by IL-1β stimulation might be due to the enhanced glycolytic metabolism in inflammatory cells, and the site where ATP was regenerated shifted from mitochondria to the cytoplasm^4,61^. *n* represents the number of biologically independent samples. *P* values are indicated in graphs and were determined using one-way ANOVA (a, b, h, i, k). Source data

In addition, CM-NTUs enhanced intracellular ATP and NADPH production in chondrocytes without IL-1β treatment (Extended Data Fig. 4a,b). We then measured the changes in ATP and NADPH levels over time in illuminated and non-illuminated cells to clarify the functional lifetime of the NTUs in cells (Extended Data Fig. 4c,d). ATP and NADPH levels in the illuminated cells gradually increased, peaked at 1–2 h and then plateaued owing to depletion of intracellular ADP and NADP^+^ pools. After 8 h, the ATP and NADPH levels began to decrease. By 32 h, ATP and NADPH levels were similar to those observed in non-illuminated cells. In non-illuminated cells, CM-NTUs had no effect on cellular ATP levels.

In addition, we examined the degradation of NTU-derived photosynthetic proteins in cells. D1 and D2 protein degradation levels were similar to those obtained by directly exposing NTUs to light (Extended Data Fig. 4e). Furthermore, the capacity of NTUs to increase cellular ATP concentrations over time under light and dark conditions were evaluated. The reduction in the ability of NTUs to increase ATP could be divided into two stages: a relatively stable period and a period of rapid decline (Extended Data Fig. 4f,g). On the basis of the results of intracellular ATP levels and photosynthetic protein degradation, we maintained an illumination time in subsequent experiments to ensure that ATP generation was relatively stable.

ROS generated by the photosynthetic apparatus did not increase the total intracellular ROS level. Moreover, intracellular ROS levels were decreased in degenerative chondrocytes containing NTUs and irradiated with light (Extended Data Fig. 4h,i). This finding may be attributed to the NADPH produced by the NTUs, which can help maintain the antioxidant enzyme (reduced glutathione) content in mammalian cells^5^.

We incubated IL-1β-treated chondrocytes with the CM-NTUs with or without light irradiation and tracked changes in chondrocytes. Treatment with CM-NTUs and light irradiation significantly increased the levels of ECM synthesis-related proteins (collagen II (Col II) and aggrecan) and decreased the levels of ECM degradation-related proteins (MMP13 and ADAMTS-5; Fig. 3d). By contrast, the CM and CM-NTUs maintained in the dark had no impact on the protein levels of chondrocytes. Increased cartilage retention was noted following treatment with the CM-NTUs and light irradiation compared with CM or CM-NTUs without light irradiation. This increase in cartilage retention was confirmed by increased *Col2a1*, *Acan* and *Sox9* mRNA levels and decreased *Mmp3*, *Mmp13* and *Adamts5* expression (Fig. 3e).

**Extended Data Fig. 5:**
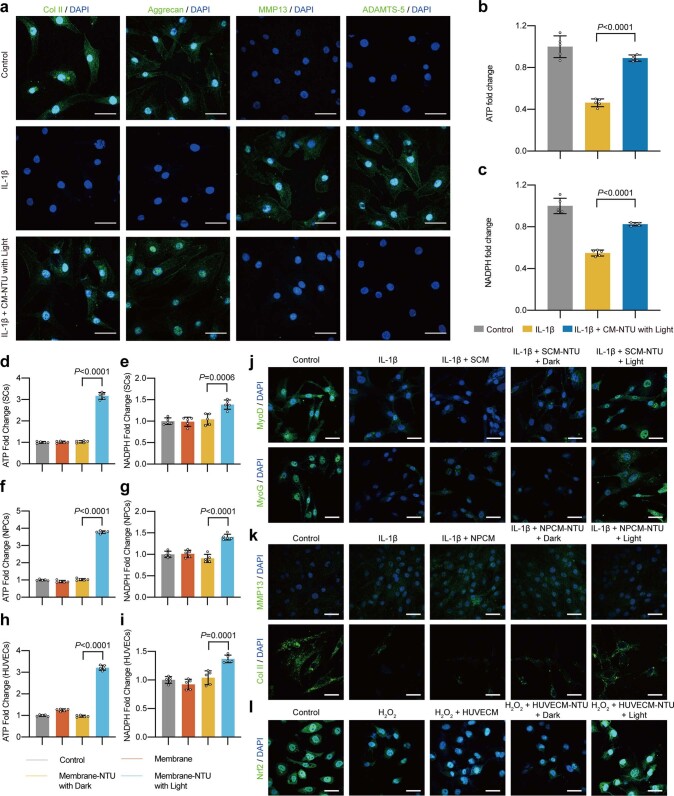
Various membrane-coated NTUs regulate intracellular metabolic homeostasis. (a) Immunofluorescence staining of Col II, aggrecan, MMP13, and ADAMTS-5 in human chondrocytes (scale bar, 10 μm). Human chondrocytes were stimulated with IL-1β for 24 h followed by CM-NTU treatment for 6 h with red light irradiation (80 µmol photons m^−2^ s^−1^, 30 min). (b, c) ATP (b) and NADPH (c) levels in human chondrocytes incubated with IL-1β or IL-1β + CM-NTUs with light (*n* = 5, mean ± SD). CM-NTUs with light can increase intracellular ATP and NADPH levels close to control levels. (d–i) ATP (d, f, h) and NADPH (e, g, i) levels in muscle satellite cells (SCs), nucleus pulposus cells (NPCs), and HUVECs. SCs, NPCs, and HUVECs were incubated with the corresponding membrane-coated NTUs with or without light irradiation (*n* = 5, mean ± SD). ATP and NADPH concentrations after light exposure were increased 3.17–3.78 and 1.37–1.40 fold those of unirradiated ATP and NADPH, respectively. (j, k) Immunofluorescence staining of myogenic markers (MyoD and MyoG) in SCs (j) and ECM markers (Col II and MMP13) in NPCs (k) (scale bar, 10 μm). SCs or NPCs were stimulated with IL-1β for 24 h followed by corresponding membrane-coated NTU treatment for 6 h with red light irradiation. (l) Immunofluorescence staining of an antioxidant marker (Nrf2) in HUVECs (scale bar, 10 μm). HUVECs were stimulated with H_2_O_2_ for 24 h followed by HUVEC membrane-coated NTU treatment for 6 h with red light irradiation. *n* represents the number of biologically independent samples. *P* values are indicated in graphs and were determined using one-way ANOVA (b–i). Source data

We tested the effect of the CM-NTUs on the dysregulation of mitochondrial activity induced by IL-1β. Significantly lower mitochondria-associated ROS levels were observed in chondrocytes treated with CM-NTUs and light exposure compared with chondrocytes treated with CM or CM-NTUs in the dark (Fig. 3f). Mitochondrial membrane potential values are used to indicate the oxidative metabolic state of cells^35^. After treatment with CM-NTUs exposed to light, the red fluorescence detected by the probe JC-1 was considerably increased compared with that after treatment with CM or CM-NTUs in the dark. The increased fluorescence was indicative of an enhanced energy state (Fig. 3f). A decreased capacity for mitochondrial biogenesis and dysfunctional energy metabolism in chondrocytes is associated with decreased sirtuin 1 (SIRT1), peroxisome proliferator-activated receptor-γ coactivator 1α (PGC1α; the so-called master regulator of mitochondrial biogenesis), transcription factor A, mitochondrial (TFAM), nuclear receptor erythroid 2-related factor 1 (NRF1) and NRF2 protein levels^24^. Chondrocytes treated with CM-NTUs and light had increased SIRT-1, PGC-1α, TFAM, NRF1 and NRF2 levels (Fig. 3g). Moreover, the cytoplasmic ATP/ADP ratio in chondrocytes decreased from 2.42 to 0.84 after IL-1β stimulation (Extended Data Fig. 4j,k). The addition of CM-NTUs restored the ratio of cytoplasmic ATP/ADP to 2.39 (Extended Data Fig. 4j,k), which indicated that the intracellular energy state was restored. We observed a similar treatment effect of the CM-NTUs in human degenerated chondrocytes (Extended Data Fig. 5a–c). These results indicate that the CM-NTUs can improve cell anabolism through a light-controllable natural photosynthetic system.

Next, we sought to evaluate the potential application of the platform for other degenerative diseases. To this end, we prepared various membrane-coated NTUs and cultured them with the corresponding cells. Enhanced ATP and NADPH levels were observed in each cell type after light irradiation (Extended Data Fig. 5d–i). Furthermore, we used IL-1β to induce degenerative changes in muscle SCs and NPCs and used H_2_O_2_ to induce oxidative stress damage in human umbilical vein endothelial cells (HUVECs). The results showed that NTUs coated with SC membrane (SCM-NTUs) exposed to light significantly increased the protein levels of myogenic markers (MyoD and MyoG) in SCs (Extended Data Fig. 5j). The NTUs coated with NPC membrane (NPCM-NTUs) and exposed to light led to considerably increased Col II protein levels and decreased MMP13 protein levels in NPCs (Extended Data Fig. 5k). Consistently, NTUs coated with HUVEC membrane (HUVECM-NTUs) and exposed to light increased the protein levels of an antioxidant marker (NRF2) in HUVECs (Extended Data Fig. 5l). These results indicate that NTUs coated with mature mammalian membrane are able to improve cell anabolism following exposure to light.

## CM-NTUs reprogramme cell metabolism

**Fig. 4:**
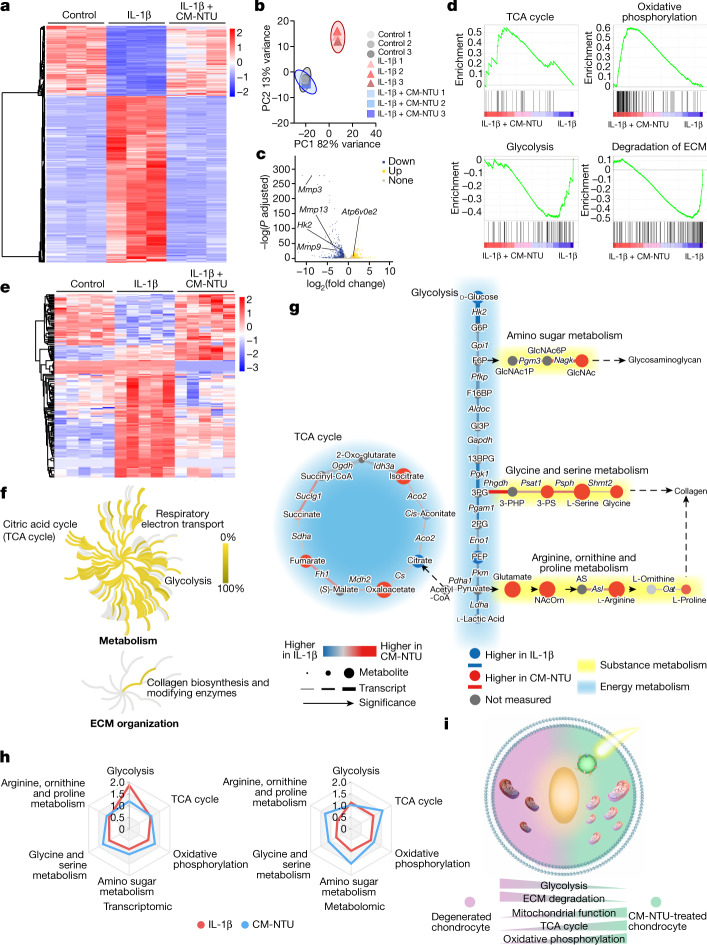
CM-NTUs promote cellular metabolic reprogramming. **a**, Heatmap showing differentially expressed genes in chondrocytes. Chondrocytes were stimulated with IL-1β followed by CM-NTU treatment for 6 h with red light irradiation (80 µmol photons m^−2^ s^−1^, 30 min). Three biological replicates are shown. **b**, PCA of genes in chondrocytes after treatment with IL-1β or with IL-1β and CM-NTUs in the light. Three replicates are shown. **c**, Volcano plots were generated representing genes related to oxidative phosphorylation, glycolysis and ECM degradation between the IL-1β plus CM-NTU group and the IL-1β group. Compared with the IL-1β group, the IL-1β plus CM-NTU group showed upregulated expression of 351 genes and downregulated expression of 784 genes (*P*-adjusted value by Wald test in DESeq2). **d**, Gene set enrichment analysis was performed to compare the gene sets involved in the TCA cycle, oxidative phosphorylation, glycolysis and ECM degradation between the IL-1β plus CM-NTU group and the IL-1β group. **e**, Heatmap representation and cluster analysis of metabolites in chondrocytes treated with IL-1β or with IL-1β and CM-NTUs in the light. Five biological replicates are shown. **f**, The influence of CM-NTU treatment on pathways related to metabolism and ECM organization. Topological graphs of these pathways are shown. Metabolomics data were analysed using the Reactome database. **g**, Concordant metabolomics integrated with transcriptomics analysis of the IL-1β plus CM-NTU with light group versus the IL-1β group. Connecting lines represent transcriptional expression of enzymes, circular nodes represent metabolite abundance and increased width indicates greater significance. **h**, Radar plot illustrating the pathway enrichment score of glycolysis, the TCA cycle, oxidative phosphorylation, amino sugar metabolism, glycine and serine metabolism, and arginine, ornithine and proline metabolism in the IL-1β group and the IL-1β plus CM-NTU groups. **i**, Schematic diagram of CM-NTU-driven metabolic reprogramming in degenerated chondrocytes.

**Extended Data Fig. 6:**
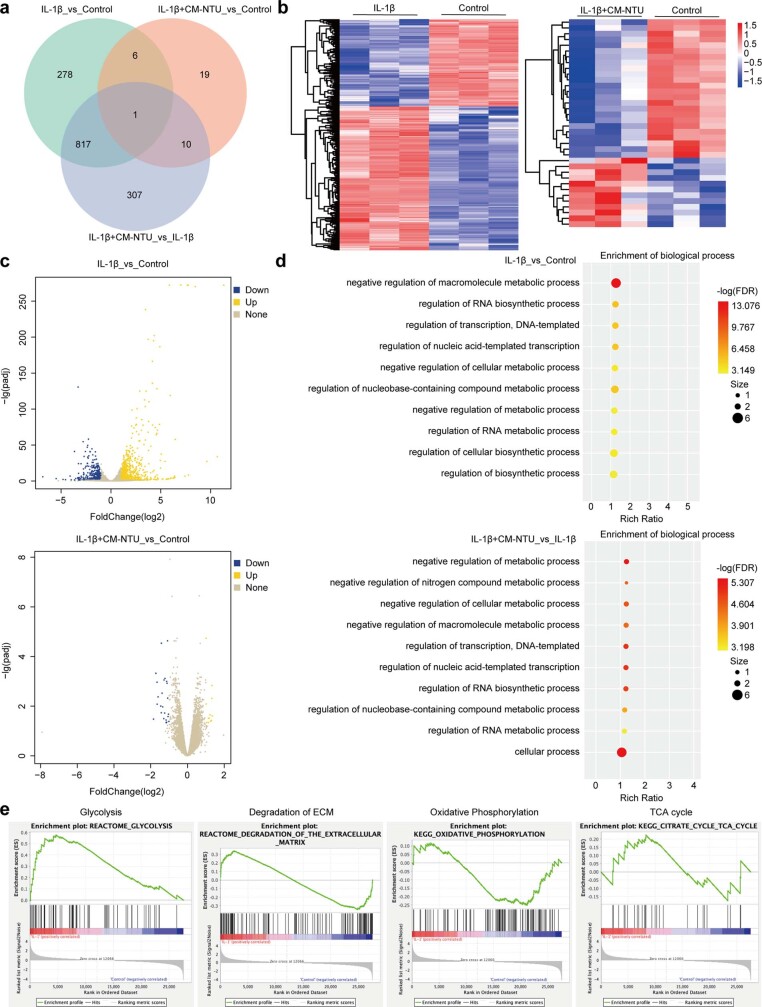
Transcriptomics study of CM-NTU-treated chondrocytes. (a) A total of 27,597 genes were identified, and 1,438 differentially expressed genes among the three groups were identified (absolute log2-fold changes ≥ 1 and *P* values < 0.05). Venn diagram for the differentially expressed genes detected in chondrocytes after treatment with IL-1β or IL-1β + CM-NTUs in the light. (b) Heatmap showing that there were fewer differentially expressed genes between the IL-1β + CM-NTU and control groups than between the IL-1β and control groups. (c) Volcano plots were generated representing differentially expressed genes between different groups (*P* adjusted value by Wald test in DESeq2). (d) GO enrichment analysis of differentially expressed genes between different groups. (e) GSEA was applied to compare the gene sets involved in glycolysis, ECM degradation, oxidative phosphorylation, and the TCA cycle between the IL-1β and control groups.

It was proposed that ATP and NADPH generation might lead to cellular metabolic enhancement. Here transcriptomics analysis was performed on chondrocytes treated with or without CM-NTUs exposed to light to comprehensively determine the changes in cell anabolism (Fig. 4a and Extended Data Fig. 6a–c). Multivariate analysis (principal component analysis (PCA)) showed that genes in the control group and the IL-1β plus CM-NTU group were colocalized in space and were highly distinct from those of the IL-1β group (Fig. 4b). GO analysis showed that the extensive genetic changes that occurred were enriched in metabolic processes (Extended Data Fig. 6d). Inflammatory stimuli may enhance the metabolic reprogramming of chondrocytes by increasing glycolysis and decreasing oxidative phosphorylation^34^. Compared with the control group, the IL-1β group showed upregulated expression of genes involved in glycolysis and downregulated expression of genes involved in oxidative phosphorylation (Extended Data Fig. 6e). We next compared gene expression patterns between the IL-1β plus CM-NTU group and the IL-1β group. The IL-1β plus CM-NTU group showed a significant increase in the expression of oxidative phosphorylation genes, such as *Atp6v0e2*, and a decrease in the expression of glycolysis and ECM degradation-related genes, such as *Hk2*, *Mmp3*, *Mmp9* and *Mmp13* (Fig. 4c). Consistently, the IL-1β plus CM-NTU group exhibited upregulated expression of genes involved in the TCA cycle and oxidative phosphorylation and downregulated expression of genes involved in glycolysis and ECM degradation (Fig. 4d).

**Extended Data Fig. 7:**
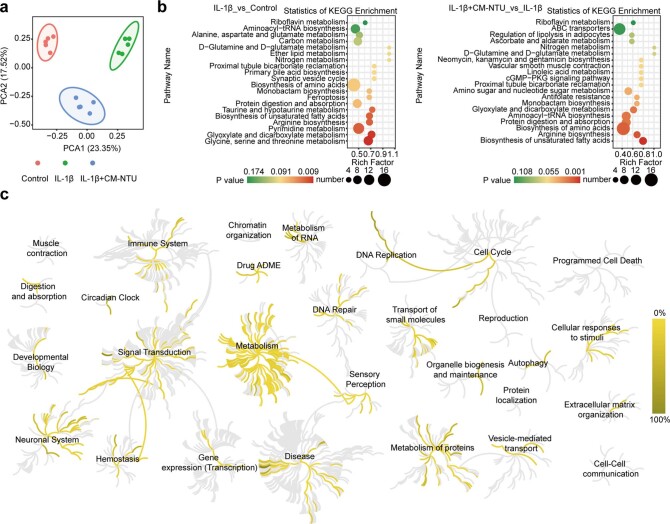
Metabolomics study of CM-NTU-treated chondrocytes. (a) Principal component analysis of metabolites in chondrocytes after treatment with IL-1β or IL-1β + CM-NTUs with light. (b) Kyoto Encyclopedia of Genes and Genomes (KEGG) enrichment analysis of the pathways involved in the biological effect induced by IL-1β or IL-1β + CM-NTU treatment. *P* values were calculated using the hypergeometric tests. (c) The Reactome data model was used to establish the equivalence of differentially abundant metabolite-related reactions across multiple networks^62^. The reaction map shows the reactions annotated in the Reactome. The reaction clusters of top-level processes are presented.

Metabolomics analysis was also performed (Supplementary Table 4). PCA indicated that the metabolites of the IL-1β plus CM-NTU group were distinct from those of the IL-1β group (Extended Data Fig. 7a). The differentially abundant metabolites were enriched in carbon and amino acid metabolism (Fig. 4e and Extended Data Fig. 7b). Networks of the 29 top‐level biological processes are displayed in Extended Data Fig. 7c. Compared with the IL-1β group, the IL-1β plus CM-NTU group showed significant effects on the cellular TCA cycle, respiratory electron transport, glycolysis and collagen synthesis (Fig. 4f).

Next, we integrated transcriptomics and metabolomics profiling to analyse cellular energy and substance metabolism^36^. Consistent with the above results, the CM-NTUs reduced glycolytic gene expression and metabolite abundance and increased TCA cycle-related gene expression and metabolite abundance (Fig. 4g,h). Cartilage ECM is mainly composed of collagen and glycosaminoglycan (GAG) and is difficult to regenerate in osteoarthritis^37,38^. We explored the changes in synthesis of these two main substances in chondrocytes. Studies have reported that glycine and serine metabolism^39^, as well as arginine, ornithine and proline metabolism^40^, promote collagen synthesis. We found that the CM-NTUs increased the metabolite abundance (l-serine, glycine, *N*-acetylornithine, l-arginine and l-proline) and gene expression (*Psat1*, *Psph*, *Shmt2*, *Asl* and *Oat*) in both mechanisms of collagen synthesis. In addition, *N*-acetyl-d-glucosamine (GlcNAc), which is involved in amino sugar metabolism, is a glucose metabolite that has a pivotal role as a key substrate in the synthesis of GAG^41^. We found that the CM-NTUs increased the levels of the metabolite GlcNAc and the expression of genes involved in GAG synthesis (*Pgm3* and *Nagk*). In summary, these results indicate that CM-NTU-driven metabolic reprogramming can systemically correct the imbalance of energy (glycolysis, TCA cycle and oxidative phosphorylation) and substance (collagen and GAG) metabolism in degenerated chondrocytes (Fig. 4i).

## Effect of CM-NTU treatment on osteoarthritis

**Fig. 5:**
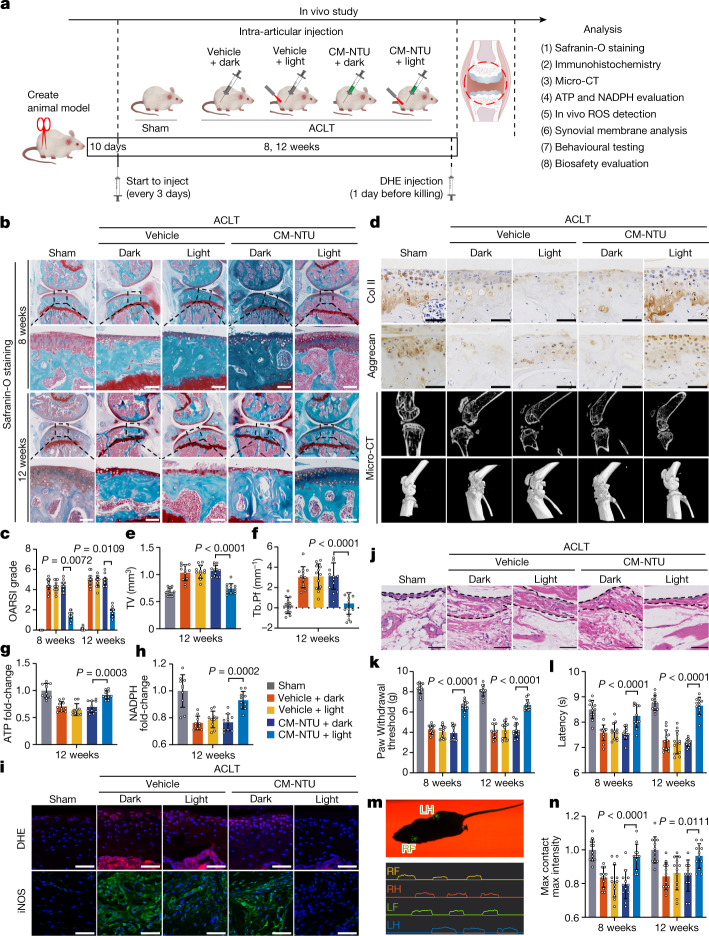
In vivo effect of CM-NTU treatment on osteoarthritis in mice. The in vivo effect of CM-NTU treatment on osteoarthritis was investigated in 12-week-old male mice. **a**, Schematic illustration of establishment of the mouse model of osteoarthritis and the experimental design to evaluate the protective effects of CM-NTUs. DHE, dihydroethidium. **b**, Safranin-O staining of joint sections at 8 and 12 weeks. **c**, Medial tibial plateau joint score based on the OARSI scoring system (*n* = 12, mean ± 95% confidence interval (CI)). **d**, Immunohistochemical staining (Col II and aggrecan) of joint sections at 12 weeks (top two rows), sagittal views of micro-CT images of the knee joints (third row) and three-dimensional images of the knee joints at 12 weeks (bottom row). **e**,**f**, Twelve weeks after the operation, quantitative analysis of total tissue volume (TV) (**e**) and trabecular pattern factor (Tb.Pf) (**f**) in subchondral bone (*n* = 12, mean ± s.d.) in mice. **g**,**h**, ATP (**g**) and NADPH (**h**) levels in CM-NTU-treated joints at 12 weeks (*n* = 10, mean ± s.d.). **i**, ROS fluorescence and immunofluorescence of iNOS in CM-NTU-treated joints at 12 weeks. **j**, H&E staining of synovial membranes in CM-NTU-treated joints at 12 weeks. **k**,**l**, Electronic von Frey (**k**) and hotplate (**l**) pain assays in mice at 8 and 12 weeks after the ACLT operation (*n* = 12, mean ± s.d.). **m**, Schematic of gait analysis. RF, right front; RH, right hind; LF, left front; LH, left hind. **n**, Gait assessment scores for maximum contact maximum intensity (right hind limb) in mice 8 and 12 weeks after operation (*n* = 12, mean ± s.d.). *n* represents the number of mice per group. *P* values are shown in graphs and were determined using nonparametric Kruskal–Wallis test (**c**) or one-way ANOVA (**e**–**h**,**k**,**l**,**n**). Scale bar, 50 μm (**d**,**i**,**j**) or 100 μm (**b**). Source data

**Extended Data Fig. 8:**
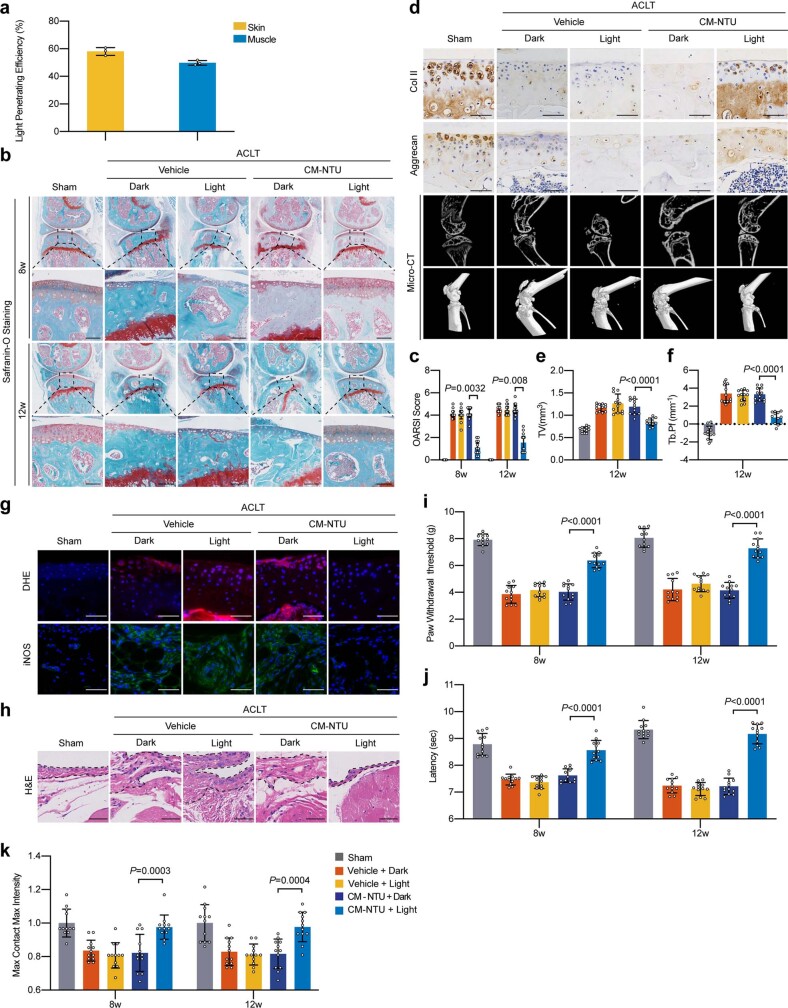
In vivo effect of CM-NTU treatment on osteoarthritis (OA) in 12-week-old female mice. (a) Efficiency of red light penetrating the skin and muscle (*n* = 3, mean ± SD). It should be noted that in the future, when applying CM-NTUs to larger human joints, the efficiency with which light penetrates human skin can be calculated first, and then the light intensity can be increased accordingly to ensure that sufficient photons reach the joint cavity. (b) Safranin O staining of joint sections at 8 and 12 weeks (scale bar, 100 μm). (c) Medial tibial plateau joint score based on the OARSI scoring system (*n* = 12, mean ± 95% CI). (d) Immunohistochemical staining (Col II and aggrecan) of joint sections at 12 weeks (scale bar, 50 μm), sagittal views of μCT of the knee joints, and three-dimensional images of the knee joints at 12 weeks. (e, f) For mice at 12 weeks after operation, quantitative analysis of TV (e) and Tb.Pf (f) in subchondral bone (*n* = 12, mean ± SD). (g) ROS fluorescence and immunofluorescence of iNOS in CM-NTU-administered joints at 12 weeks (scale bar, 50 μm). (h) H&E staining of synovial membranes in CM-NTU-treated joints at 12 weeks (scale bar, 50 μm). (i, j) Electronic von Frey (i) and hot-plate (j) pain assays in mice at 8 and 12 weeks after the operation (*n* = 12, mean ± SD). (k) Gait assessment scores for maximum contact maximum intensity (right hind limb) in mice at 8 and 12 weeks after operation (*n* = 12, mean ± SD). *n* represents the number of mice per group. *P* values are indicated in graphs and were determined using the nonparametric Kruskal–Wallis test (c) or one-way ANOVA (e, f, i–k). Source data

Owing to the closed environment of the knee joint cavity, we first measured the efficiency of red light to penetrate skin and muscle. Values of 58.1% and 49.8%, respectively, were achieved (Extended Data Fig. 8a), which demonstrates that red light can effectively penetrate the knee joint cavity in our model. Next, we investigated whether intra-articular injection of the CM-NTUs and subsequent exposure to light irradiation can inhibit the progression of osteoarthritis induced by anterior cruciate ligament transection (ACLT) surgery in mice (Fig. 5a). CM-NTU treatment in combination with light irradiation significantly attenuated cartilage destruction (as assessed by safranin-O staining) at 8 and 12 weeks after surgery (Fig. 5b). The Osteoarthritis Research Society International (OARSI) scores further confirmed this result. Compared with ACLT controls, the scores of mice subjected to ACLT and treated with CM-NTUs and light were significantly reduced (1.45 and 1.81 at 8 and 12 weeks after surgery, respectively). These results indicated that the knee joints treated with CM-NTUs and light exhibited fewer osteoarthritic changes compared with knees exposed to other treatments (Fig. 5c). Immunohistochemistry performed on sections of joint showed increased Col II and aggrecan content after CM-NTUs and light treatment (Fig. 5d).

Next, we used microcomputed tomography (micro-CT) to examine the tibial subchondral bone medial compartment^42^. The results showed that mice treated with CM-NTUs and light exhibited reduced morphological alterations of the subchondral bone plate and osteophyte formation at 12 weeks after surgery (Fig. 5d). In addition, quantitative analysis indicated that treatment with CM-NTUs and light inhibited tibial subchondral bone remodelling, with reduced total tissue volume and trabecular pattern factor (Fig. 5e,f).

Analyses of ATP and NADPH concentrations of articular cartilage in each group showed that CM-NTUs plus light increased the production of ATP and NADPH (Fig. 5g,h). Evaluation of the in vivo ROS level in frozen sections of joint tissues at 12 weeks using dihydroethidium was next performed. After ACLT surgery, the fluorescence intensity of ROS increased in the articular cartilage, and treatment with CM-NTUs and light reversed the generation of ROS in the cartilage microenvironment (Fig. 5i). Inflammation of the synovial membrane is an important feature of osteoarthritis^43^. Treatment with CM-NTUs and light reduced the protein levels of inducible nitric oxide synthase (iNOS) (Fig. 5i). Haematoxylin and eosin (H&E) staining revealed that treatment with CM-NTUs and light reduced hypertrophy and hyperplasia of the synovium and decreased the thickness of the synovial lining cell layer (Fig. 5j).

**Extended Data Fig. 9:**
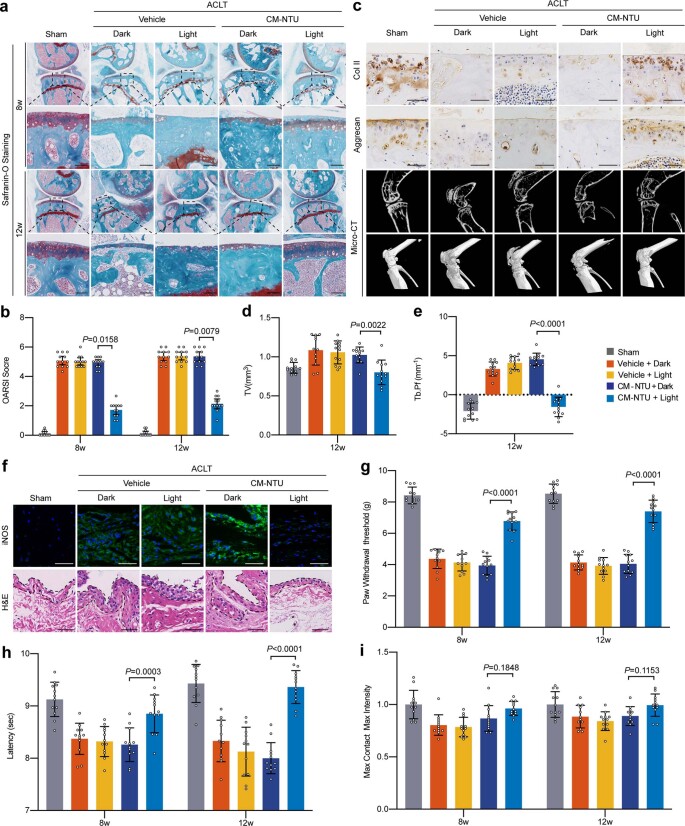
In vivo effect of CM-NTU treatment on osteoarthritis (OA) in 12-month-old male mice. (a) Safranin O staining of joint sections at 8 and 12 weeks (scale bar, 100 μm). (b) Medial tibial plateau joint score based on the OARSI scoring system (*n* = 12, mean ± 95% CI). (c) Immunohistochemical staining (Col II and aggrecan) of joint sections at 12 weeks (scale bar, 50 μm), sagittal views of μCT of the knee joints, and three-dimensional images of the knee joints at 12 weeks. (d, e) For mice at 12 weeks after operation, quantitative analysis of TV (d) and Tb.Pf (e) in subchondral bone (*n* = 12, mean ± SD). (f) Immunofluorescence of iNOS and H&E staining of synovial membranes in CM-NTU-administered joints at 12 weeks (scale bar, 50 μm). (g, h) Electronic von Frey (g) and hot-plate (h) pain assays in mice at 8 and 12 weeks after the operation (*n* = 12, mean ± SD). (i) Gait assessment scores for maximum contact maximum intensity (right hind limb) in mice at 8 and 12 weeks after operation (*n* = 12, mean ± SD). *n* represents the number of mice per group. *P* values are indicated in graphs and were determined using the nonparametric Kruskal–Wallis test (b) or one-way ANOVA (d, e, g–i). Source data

**Extended Data Fig. 10:**
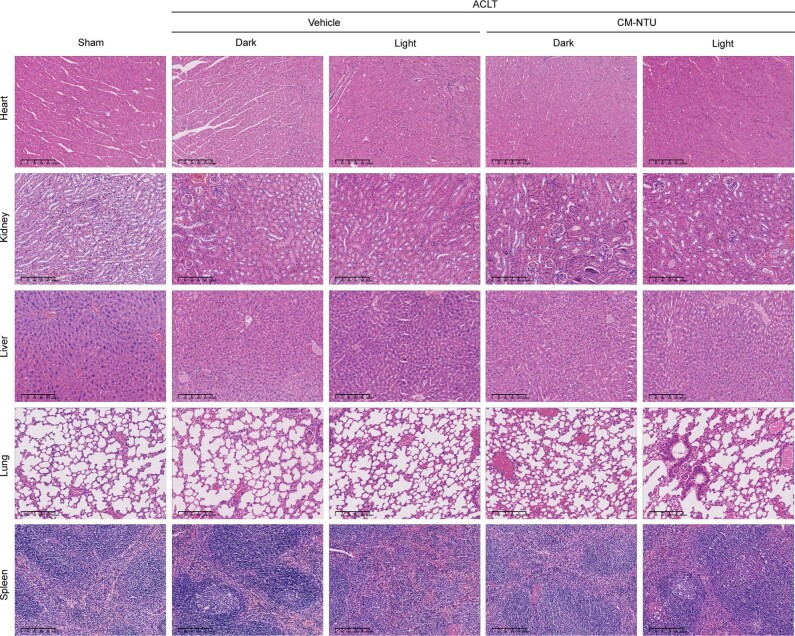
In vivo safety of CM-NTUs in 12-week-old male mice. H&E staining of histological sections from major organs, including the heart, liver, spleen, lungs, and kidneys (scale bar, 200 μm; *n* = 12 mice per group). From H&E staining, we observed that the overall structure, integrity, and immune infiltrate levels in heart, liver, spleen, lung, and kidney tissues in the CM-NTU-administered mice were nearly identical to the those from the sham controls. Data are representative of two independent experiments.

CM-NTU plus light irradiation also reduced ACLT-induced osteoarthritis pain (Fig. 5k,l). Gait analysis showed that the maximum contact and maximum intensity of the right hind limb of mice in the ACLT control group were significantly reduced compared with that in the sham group, and this effect was rescued in ACLT mice treated with CM-NTUs and light (Fig. 5m,n). We repeated the treatment experiment in 12-week-old female mice and in 12-month-old male mice. The CM-NTUs and light treatment led to similar effects, in that mice showed substantial reductions in ACLT-induced post-traumatic osteoarthritis and related pain (Extended Data Figs. 8 and  9). Histological analyses of major organs confirmed the biosafety of CM-NTU treatment in vivo (Extended Data Fig. 10). Taken together, this study demonstrates that CM-NTUs can promote cartilage homeostasis and protect against osteoarthritis progression in animals.

In summary, using a membrane-coating strategy, we demonstrated the feasibility and applicability of cross-species transplantation of a plant-derived natural photosynthetic system. This treatment strategy is generalizable as coatings derived from different mature mammalian cell sources can be used to provide application-specific benefits for various degenerative diseases. Moreover, this strategy can overcome the limitation of elimination and rejection by the body. In addition, we constructed a completely natural photosynthesis system that can independently facilitate the supply of key energy and metabolic carriers in cells based on exposure to light. Finally, this study provides new methods for the precise regulation of metabolism using natural materials. This photosynthesis system can enhance cell anabolism and exhibits promising clinical potential in the treatment of degenerative diseases.

## Methods

### Materials

RIPA lysis buffer, BSA, EDTA, Tris-HCl, protease inhibitor cocktail, Coomassie blue, 3,3′-dioctadecyloxacarbocyanine perchlorate (DiO), 1,1′-dioctadecyl-3,3,3′,3′-tetramethylindodicarbocyanine perchlorate (DiD) and DiI were purchased from Dalian Meilun Biotechnology. Lecithin was purchased from Solarbio, cholesterol was purchased from Shanghai Yuanye BioTechnology, and 2-distearoyl-*sn*-glycero-3-phos-phoethanolamine-N-[methoxy(polyethylene glycol)-2000] (DSPE-PEG2000) was purchased from AVT. Ferredoxin and FNR from *Spinacia oleracea* were purchased from Sigma-Aldrich. All other chemicals were purchased from Sigma-Aldrich unless specifically mentioned.

### NTU preparation

Thylakoids were isolated from young spinach leaves using a modified method^13^. The obtained thylakoids were pooled, diluted and sonicated for 2 min in a Fisher Scientific FS30D bath sonicator. This step was followed by extrusion through 100-nm polycarbonate porous membranes (Whatman) using an Avanti mini extruder. The solutions were then centrifuged for 60 min at 100,000*g*. The pellet was resuspended in osmotic shock buffer (10 mM HEPES-KOH, 10 mM MgCl_2_ and 10 mM sodium l-ascorbate). NanoSight NS300 (Malvern Instruments) was used to detect the concentration (particles per ml) of NTUs. The NTUs were flash-frozen with 10% DMSO as an osmoprotectant and stored at −80 °C until use. Before use, the NTUs were stored on ice and washed two to three times in osmotic shock buffer. A similar method was applied to encapsulate gold nanoparticles into the NTUs, and equal volumes of gold nanoparticles and thylakoids were mixed and then sonicated and extruded. The chlorophyll content of the resulting solution was determined using a chlorophyll assay kit (Acmec).

### Assays of NTU activity

We tested the independent photosynthesis capacity of the NTUs to synthesize ATP and NADPH in vitro using a previous method^13^ to construct a reaction system in vitro. In a reaction volume of 0.7 ml, NTUs were added to a reaction buffer containing 50 mM HEPES-KOH pH 7.8, 5 μM ferredoxin, 3 mM ADP, 5 mM K_2_HPO_4_, 3 mM NADP^+^, 10 mM sodium l-ascorbate, 10 mM KCl, 5 mM MgCl_2_, 1.5 μM catalase and 52 U ml^−1^ bovine superoxide dismutase and illuminated with red actinic light from light-emitting diodes, peaking at 630 nm at an intensity of 80 µmol photons m^−2^ s^−1^. Samples were obtained at 0, 5, 10 and 15 min, and the ATP concentration was measured using an ATP assay kit (Beyotime). For NADPH measurement, samples were illuminated with red light for 10 min, and NADPH production was measured every 5 min using a NADPH assay kit (Colorimetric, Abcam). To test the stability of NTUs, the abundance of D1 and D2 proteins (susceptible to photooxidation damage^44–46^) in NTUs over time under light and dark conditions was detected by western blotting. The prepared NTUs were illuminated at room temperature. The changes in ATP production capacity of the NTUs over time were measured under light and dark conditions.

### Membrane-coated NTU preparation

Cell membranes were collected from chondrocytes according to a previously published protocol^47^. The membrane was suspended at 2 mg ml^−1^ in water. The proteins in the membrane were subsequently assessed by western blotting to detect Na^+^/K^+^-ATPase (a membrane-specific marker) and β-tubulin (a plasma-specific marker). CM solution was then added to an equal volume of NTUs for 30 min followed by sequential extrusion through 1,000, 400 and 200 nm polycarbonate porous membranes (Whatman) using an Avanti mini extruder. CM-NTUs were isolated by centrifugation at 10,000*g* for 5 min and then resuspended in water for further use. For CM-NTUs used in cellular NADPH experiments, different concentrations of ferredoxin (0–250 μM) were encapsulated into CM-NTUs in the extrusion process. The estimate of the ferredoxin dilution ratio (about 21-fold) was based on the ratio of the volume of CM-NTUs delivered into the cell (around 190 μm^3^; particle sizes of NTUs and CM-NTUs are approximately 130 nm and 216 nm, respectively, and the thickness of the cell membrane is about 6 nm^48^) and the total volume of the cell cytoplasm (around 3,800 μm^3^; cells with a diameter of 20 μm and a nucleus volume of 10% of the cell volume^48^). CM vesicles were prepared by extruding purified CM through the same set of porous membranes. Other cell-derived membrane-coated NTUs were prepared in a similar manner.

For LNPs, 20 mg of DSPE-PEG2000, 100 mg of lecithin and 16 mg of cholesterol were dissolved in 10 ml of CHCl_3_. When the organic solvent was evaporated, a thin lipid film was generated on the inner wall of the flasks. The film was hydrated and sonicated to obtain the LNPs. To produce LNP-NTUs, the lipid film was added with an equal volume of NTUs for 30 min followed by sequential extrusion through 1,000, 400 and 200 nm polycarbonate porous membranes. The LNP-NTUs were isolated by centrifugation at 10,000*g* for 5 min and then resuspended in water for further use.

### Nanoparticle characterization

Nanoparticle size and surface zeta potential were measured by dynamic light scattering using a Malvern Zetasizer Nano ZS^49^. Nanoparticle morphology was observed by cryo-TEM (200 kV, FEI Tecnai G2 F20) or TEM (H-9500, Hitachi).

### Proteomics analysis

To study whether the NTUs have an independent photosynthetic function of the thylakoid organelle and to analyse the biological functions (homotypic targeting and membrane fusion^47,50^) of CM proteins, we analysed the NTUs and CM using proteomics. Protein from the NTUs and CM was analysed according to a previously published protocol^51^. In brief, consecutive fractions were collected for liquid chromatography–tandem mass spectrometry analysis. To determine the biological and functional properties of all identified proteins, the identified protein sequences were analysed on the basis of GO terms.

### Cell culture

RAW 264.7 mouse macrophages, a HUVEC line and a mouse fibroblast cell line (NIH/3T3) were obtained from the China Center for Type Culture Collection. For primary chondrocytes, mouse articular cartilage was dissected from the knee joint of 4-week-old male C57BL/6 mice. Human cartilage was obtained from human participants without osteoarthritis. The study design and protocol were approved by the ethics committee of Sir Run Run Shaw Hospital. Informed consent was obtained. Chondrocytes were obtained by overnight digestion of cartilage pieces with 0.025% Col II (Roche Diagnosis). To obtain primary NPCs, nucleus pulposus tissue was macroscopically isolated from 4-week-old male Sprague–Dawley rats. Next, the tissues were diced into small pieces and treated with 0.025% Col II at 37 °C for 4 h. NPCs were obtained after resuspension and filtration. To obtain primary muscle SCs, gastrocnemius muscle from 4-week-old male C57BL/6 mice was minced and digested with 5 mg ml^−1^ collagenase IV (Gibco-Thermo Fisher) and 1.2 U ml^−1^ dispase (Gibco-Thermo Fisher) at 37 °C for 45 min. SCs were obtained after resuspension and filtration. Primary cells were maintained as a monolayer in DMEM supplemented with 10% FBS. Second-passage cells were used for the subsequent experiments.

### Antibodies

For western blot analysis, the following antibodies were used: D1 (Agrisera, AS05084; 1:10,000); D2 (Agrisera, AS06146; 1:5,000); β-tubulin (Abcam, ab179511, clone EPR16778; 1:1,000); Na^+^/K^+^-ATPase (Abcam, ab76020, clone EP1845Y; 1:20,000); SIRT1 (Proteintech, 13161-1-AP; 1:1,000); PGC1a (Proteintech, 66369-1-Ig, clone 1C1B2; 1:5,000); TFAM (Proteintech, 22586-1-AP; 1:5,000); NRF1 (Proteintech, 12482-1-AP; 1:500); NRF2 (Proteintech, 16396-1-AP, 1:1,000); β-actin (Proteintech, 20536-1-AP; 1:1,000); AtpB (Agrisera, AS05085; 1:2,000); anti-rabbit IgG HRP-linked secondary antibody (FDbio science, FDR007; 1:5,000); and anti-mouse IgG HRP-linked secondary antibody (FDbio science, FDM007; 1:5,000). For immunofluorescence analysis, the following antibodies were used: Col II (Proteintech, 28459-1-AP; 1:800); aggrecan (Proteintech, 13880-1-AP; 1:200); MMP13 (Proteintech, 18165-1-AP; 1:200); ADAMTS-5 (Abcam, ab246975; 1:500); iNOS (Abcam, ab178945, clone EPR16635; 1:500); MyoD (Proteintech, 18943-1-AP; 1:200); MyoG (Abcam, ab124800, clone EPR4789; 1:500); NRF2 (Proteintech, 16396-1-AP; 1:200); and CoraLite488-conjugated goat anti-rabbit IgG (Proteintech, SA00013-2; 1:500). For immunohistochemistry analysis, the following antibodies were used: Col II (Proteintech, 28459-1-AP; 1:800); aggrecan (Proteintech, 13880-1-AP; 1:200); and goat anti-rabbit IgG secondary antibody (Thermo Fisher, 31460; 1:500).

### Cell viability assay

Chondrocytes were seeded into 96-well plates at a density of 4,000–5,000 cells per well and incubated for 24 h followed by the addition of the treatments at the indicated concentrations (2 × 10^5^ NTUs per cell). After an additional 24, 48 or 72 h of incubation, cell viability was measured using a Cell Counting Kit-8 (CCK-8) assay following the manufacturer’s instructions (Dojindo).

### Cellular uptake of NTUs

We sought to characterize the cross-species impact of mammalian cell membrane coating on plant-derived photosynthetic organelle interactions with macrophages and mature tissue cells (chondrocytes). RAW 264.7 mouse macrophages and mouse chondrocytes were used for the cellular uptake experiments. Cells were incubated in 12-well plates (1 × 10^5^ cells per well) and cultured for 1 day. The NTUs were labelled with DiI before coating with LNPs or CM. Then, DiI-labelled NTUs, LNP-NTUs and CM-NTUs were used at a concentration of 2 × 10^5^ NTUs per cell to study the cellular internalization efficiency. RAW 264.7 mouse macrophages were incubated with the NTUs for 6 h. Chondrocytes were incubated with NTUs for 1, 3 and 6 h. The cell samples were washed three times with PBS for 5 min and fixed with 4% polyformaldehyde (PFA) for 20 min. Then, the cells were stained with 4′,6-diamidino-2-phenylindole (DAPI) for 20 min at 25 °C to label nuclei. Finally, the cells were observed by laser confocal microscopy (LCM; Nikon) or structured illumination microscopy (Nikon). The DiI fluorescence signal was measured using a Synergy H4 hybrid microplate reader (Bio Tek). A fluorescence-based assay was performed to estimate the numbers of NTUs delivered to each cell.

### Membrane fusion of CM-NTUs

Chondrocytes were incubated in a 96-well plate and labelled with DiI, and the outer membrane of the CM-NTUs was labelled using DiO. The CM-NTUs were then incubated with the chondrocytes at 37 °C for 1 h. The samples were fixed with 4% PFA and stained with DAPI. The images were captured and analysed by LCM. In a parallel experiment, we first labelled the NTUs with DiO and encapsulated them with CM (labelled with DiI). Then, the CM-NTUs were incubated with chondrocytes at 37 °C for 1 h before DAPI staining and LCM observation.

### Effects of endocytosis inhibitors on the cellular uptake of NTUs

We applied endocytosis-related inhibitors to study the cellular uptake pathway. Sufficient chondrocytes were seeded in 12-well plates to reach 60–70% confluency after overnight incubation. The medium was replaced with fresh medium, and four endocytosis inhibitors (chlorpromazine, filipin III, wortmannin and cytochalasin D) were subsequently separately added to the medium at concentrations of 50, 7.5, 5 or 5 µM. In particular, chlorpromazine, filipin III, wortmannin and cytochalasin D inhibited clathrin-dependent endocytosis, caveolae-dependent endocytosis, macropinocytosis and phagocytosis, respectively. After 30 min of preincubation, the cells were treated with DiI-labelled CM-NTUs (2 × 10^5^ NTUs per cell) in the presence of the inhibitors for an additional 6 h. Finally, the cells were trypsinized, isolated by centrifugation and resuspended in PBS. The fluorescence intensity in each well was quantitatively determined by flow cytometry (FACSCalibur). FlowJo (v.10) was used for flow cytometry analysis.

### Selectivity of chondrocytes taking up CM-NTUs

Equal amounts (1 × 10^5^ cells) of chondrocytes (Hoechst 33342-labelled nuclei and DiI-labelled cell membranes), NPCs (Hoechst 33342-labelled nuclei and DiD-labelled cell membranes), SCs (DiI-labelled cell membranes), macrophages (DiD-labelled cell membranes) and fibroblasts (Hoechst 33342-labelled cell nuclei) were cultured on Petri dishes and incubated overnight. CM-NTUs (DiO-labelled NTUs) at a concentration of 2 × 10^5^ NTUs per cell were added and incubated with these cells for 6 h. Then, flow cytometry was performed. In another experiment, five types of cell membrane-coated NTUs in equal amounts (2 × 10^5^ NTUs per cell) were incubated with 2 × 10^5^ chondrocytes. Owing to the limited types of staining labels, two staining schemes were used in two parallel experiments. In the first experiment, chondrocyte nuclei were labelled with Hoechst 33342. The following staining schemes were established with five different membrane-coated NTUs, including CM-NTUs (NTUs labelled with DiO), NPCM-NTUs (NTUs labelled with DiI), MM-NTUs (macrophage membrane-NTUs; NTUs labelled with DiD), SCM-NTUs (unlabelled) and FM-NTUs (fibroblast membrane-NTUs; unlabelled). These five materials were added to the culture medium and cultured with chondrocytes for 6 h (scheme 1). In the second experiment, NPCM-NTUs and MM-NTUs were not labelled, SCM-NTUs and FM-NTUs were labelled with DiI and DiD, and the remainder remained unchanged (scheme 2). Then, flow cytometry analysis was performed (LSRFortessa).

### Intracellular trafficking of NTUs in chondrocytes

To demonstrate that the CM-NTUs could avoid lysosomal elimination in mammalian cells, we stained the cells with a lysosomal marker. Chondrocytes were seeded at a density of 1.5 × 10^5^ cells per Petri dish and incubated for 24 h. The medium was replaced with fresh medium containing LNP-NTUs or CM-NTUs (NTUs labelled with DiI) at a concentration of 2 × 10^5^ NTUs per cell. The cells were then incubated for an additional 6 h. Then, lysosomes were labelled with LysoTracker Green (200 nM) for 0.5 h according to the manufacturer’s instructions, and the nuclei were stained with DAPI for 20 min. The images were captured and analysed by LCM.

### Comparison of the penetration of the LNP-NTUs and CM-NTUs in cartilage explants

Human cartilage was obtained from patients with osteoarthritis undergoing total knee replacement. The study design and protocol were approved by the ethics committee of Sir Run Run Shaw Hospital. Informed consent was obtained. Cartilage explants were extracted as solid cylinders using a sterilized 6.4-mm perforator. The explants were washed in DMEM and placed in a 96-well plate with fresh DMEM containing LNP-NTUs or CM-NTUs (NTUs labelled with DiI). After culture for 24 h, the explants were collected, immediately frozen and sectioned in a cryostat (10 μm thick). The images were captured and analysed by LCM.

### Effects of EV secretion inhibitors on CM-NTU penetration

To explore the mechanism by which the CM-NTUs can achieve deep penetration, the neutral sphingomyelinase‐targeting inhibitor GW4869 (Sigma–Aldrich), which can inhibit EV secretion, was used. The prepared cartilage explants were pretreated with 10 µM GW4869 for 24 h. Afterwards, CM-NTUs (NTUs labelled with DiI) were added to the well and incubated for 24 h. Then, the explants were removed, washed with PBS and observed by LCM.

### Secretion of CM-NTUs by chondrocytes

Chondrocytes were seeded at a density of 1.5 × 10^5^ cells per Petri dish and incubated overnight. To inhibit EV secretion, chondrocytes were pretreated with 10 µM GW4869 for 24 h. Then, LNP-NTUs or CM-NTUs (NTUs labelled with DiI) were added to the dish at a concentration of 2 × 10^5^ NTUs per cell and incubated for 1 h. Afterwards, the cells were rinsed with PBS three times and incubated with fresh medium. The culture medium was changed every few hours for 48 h. At timed intervals, the DiI fluorescence signal in the cells and culture medium was measured using a Synergy H4 hybrid microplate reader (Bio Tek).

### Transcellular delivery of CM-NTUs

Chondrocytes were seeded on coverslips (1) or (2) and incubated overnight. To inhibit EV secretion, chondrocytes were pretreated with 10 µM GW4869 for 24 h. The cells on coverslips (1) were first cultured with CM-NTUs or LNP-NTUs (NTUs labelled with DiI) at a concentration of 2 × 10^5^ NTUs per cell for 6 h. The cells on coverslips (1) were rinsed with PBS three times and then incubated with fresh cells on coverslips (2) in fresh medium for 24 h. Afterwards, the cells were washed with PBS and stained with DAPI before imaging by LCM.

### ATP and NADPH enhancement in various types of cells

The general applicability of membrane-coated NTUs was studied using several types of mammalian cells, including chondrocytes, SCs, NPCs and HUVECs. The fold-changes in ATP and NADPH levels in various cell types were measured immediately after 6 h of CM-NTU, SCM-NTU, NPCM-NTU or HUVECM-NTU (2 × 10^5^ NTUs per cell) incubation followed by 30 min of red light irradiation (80 µmol photons m^−2^ s^−1^). The intracellular ATP and NADPH concentrations were measured using assay kits, and fold-changes in ATP and NADPH were compared. To clarify the function of the NTUs in cells over time, the changes in ATP and NADPH levels over time (0–32 h) in illuminated and non-illuminated chondrocytes were measured. The degradation of NTU-derived proteins (D1, D2 and AtpB) in chondrocytes was detected by western blotting. Changes in the capacity of the NTUs to increase cellular ATP concentrations were evaluated over time under light and dark conditions. To clarify whether NTUs cause a cellular stress response, the production of ROS in cells containing NTUs under different red light illumination conditions (8.9–320 µmol photons m^−2^ s^−1^) was tested by flow cytometry (FACSCalibur) using the membrane-permeable fluorescent probe dichlorodihydrofluorescein diacetate (DCFH-DA, Beyotime).

### Membrane-coated NTUs improve cell anabolism

IL-1β is highly correlated with an increase in ECM degradation and dysregulation of mitochondrial activity and associated with increased ROS generation, reduced mitochondrial biogenesis and decreased mitochondrial ATP generation in osteoarthritis^24^. Chondrocytes, SCs and NPCs were stimulated with IL-1β (10 ng ml^−1^) for 24 h followed by corresponding cell membrane-coated or membrane-coated NTUs (2 × 10^5^ NTUs per cell) treatment for 6 h with or without red light irradiation (80 µmol photons m^−2^ s^−1^, 30 min). For chondrocytes, the levels of ECM synthesis-related proteins (Col II and aggrecan) and ECM degradation-related proteins (MMP13 and ADAMTS-5) were measured by immunofluorescence staining. JC-1 dye (Invitrogen) and MitoSOX-Red fluorescent probes (Life Technologies) were used to determine mitochondrial membrane potential and mitochondria-associated ROS production in chondrocytes, respectively, according to the manufacturer’s instructions. SIRT1, PGC1α, TFAM, NRF1 and NRF2 protein levels were detected by western blotting. The cytoplasmic ATP/ADP ratio was detected using a genetically encoded fluorescent biosensor of adenylate nucleotides (PercevalHR, a gift from G. Yellen^52^). For SCs, the protein levels of myogenic markers (MyoD and MyoG) were measured by immunofluorescence staining. For NPCs, the protein levels of Col II and MMP13 were measured by immunofluorescence staining. HUVECs were stimulated with 500 μM H_2_O_2_ for 24 h to induce oxidative stress damage followed by treatment with HUVEC-NTU (2 × 10^5^ NTUs per cell) for 6 h with or without red light irradiation (80 µmol photons m^−2^ s^−1^, 30 min). Then, the protein levels of an antioxidant marker (NRF2) were measured by immunofluorescence staining.

### Quantitative PCR

Total RNA was isolated from cells using a RNA kit (Qiagen). The RNA was reverse-transcribed into cDNA with reverse transcription reagents (Promega). Here, quantitative PCR (qPCR) and qPCR with reverse transcription systems (A6002, Promega) were used according to the manufacturer’s instructions. *Gapdh* was used as a reference gene to normalize other genes. A list of the primer sequences used for qPCR in this study is provided in Supplementary Table 5.

### Western blot analysis

RIPA lysis buffer was used for protein extraction. Then, SDS–PAGE gels were used to separate the extracted protein. After electrophoresis, polyvinylidene difluoride membranes were used for protein transfer. The proteins were then blocked with nonfat milk. After incubation with primary and secondary antibodies, a chemiluminescent signal was achieved using detection reagents (enhanced chemiluminescence; Beyotime).

### Transcriptomics and metabonomics study

For the transcriptomics study, 2 μg RNA per sample was used as input material for the RNA sample preparations. Sequencing libraries were generated using a NEBNext Ultra RNA Library Prep kit for Illumina (E7530L, NEB) following the manufacturer’s recommendations, and index codes were added to attribute sequences to each sample. Genes with *P* < 0.05 and absolute log_2_(fold changes) ≥ 1 were identified as differentially expressed genes. GO enrichment analysis of differentially expressed genes was implemented using the hypergeometric test. To determine the level of metabolic pathway enrichment, we used gene set enrichment analysis (GSEA) to compare the pathways between different groups^53^. The complete transcriptome of all samples was used for GSEA, and only gene sets with nominal *P* < 0.05 and false discovery rate *q* values < 0.06 were considered significant.

For the metabonomics study, cells were collected according to the manufacturer’s instructions, and the sample extracts were analysed using an LC–ESI–MS/MS system (ultra-performance liquid chromatography, ExionLC AD System; mass spectrometry, QTRAP System)^54^. Metabolite quantification and further analysis were performed using a multiple reaction monitoring method. Metabolites with *P* < 0.05 and fold change > 10% were deemed to be significant. A previously described network analysis pipeline^36^ was used to construct the integrated transcriptomics and metabolomics map.

### Induction of osteoarthritis and intra-articular injection of CM-NTUs

Experimental osteoarthritis was induced in C57BL/6 mice. For each experiment, sex- and age-matched mice were used and randomly allocated to each experimental group. The injections were performed by blinded investigators. All animal studies were performed according to ethical regulations and protocols approved by the Sir Run Run Shaw Hospital Committee for Animal Resources and the Institutional Animal Care and Use Committee of Zhejiang Center of Laboratory Animals. All mouse experimental procedures were performed following the Regulations for the Administration of Affairs Concerning Experimental Animals approved by the State Council of People’s Republic of China. Animals were housed in groups of 4–6 mice per individually ventilated cage in a 12-h light–dark cycle (6:30–18:30 light; 18:30–6:30 dark) with constant room temperature (21 ± 1 °C) and relative humidity (40–60%). Animals had access to food and water ad libitum. A laser power metre (Zhongxi Equipment) was used to measure the efficiency of red light penetrating the mouse skin and muscle (3 mm thickness), and 3 C57BL/6 mice (8 weeks old, male) were used in the experiment.

We determined the minimum number of animals required for a specific study based on previous experiments in our group or in the published literature. Prospective power analysis was performed using G*Power analysis. The probability values of type I and type II errors were set at 0.05 and 0.20, respectively. The power analysis showed that at least 8 mice in each group were needed. We increased the number of mice to 12 per group. Three different types of mice (12-week-old male mice, 12-week-old female mice and 12-month-old male mice) were used for the treatment studies. For the surgically induced osteoarthritis mouse model, we anaesthetized mice with ketamine and xylazine and then surgically transected the anterior cruciate ligament to induce mechanical instability-associated osteoarthritis in the right knee. Control mice were sham-operated with the anterior cruciate ligament visible but not transected. Mice were randomly divided into five groups: sham surgery (sham), transection and 20 μl vehicle (PBS) treatment every 3 days without light irradiation (ACLT + vehicle + dark); transection and 20 μl vehicle treatment every 3 days with red light irradiation for 30 min every day (ACLT + vehicle + light); transection and CM-NTUs (2 × 10^10^ NTUs) treatment every 3 days without light irradiation (ACLT + CM-NTUs + dark); and transection and CM-NTUs (2 × 10^10^ NTUs) treatment every 3 days with red light irradiation in the knee joint for 30 min every day (ACLT + CM-NTUs + light). Treatments were administered by intra-articular injection into the affected joint 10 days after surgery. By quantification of ATP content in the whole femoral and tibial articular surface isolated from mice, we estimated the total number of chondrocytes in a single mouse knee joint to be about 1 × 10^5^, and we injected 2 × 10^10^ NTUs (coated by CM) into each joint based on the dose of cell experiments. In the mouse cohorts (12-week-old male mice) used for ATP and NADPH analysis, tissues from the whole femoral and tibial articular surfaces were isolated and identified with *n* = 10 per group based on power analysis using preliminary data. At week 8 or 12, the mice were euthanized, and the joint was collected for assessment.

### In vivo micro-CT image analysis

For micro-CT analysis, samples were first fixed with 4% PFA for 48 h. The knee joints were analysed using high-resolution micro-CT (Skyscan1275). We defined the region of interest to cover the entire tibial subchondral bone medial compartment. The three-dimensional structural parameters analysed included total tissue volume (containing both trabecular and cortical bone) and trabecular pattern factor.

### Histological analysis and immunostaining

Knee joint samples were fixed and decalcified before histological analysis. Subsequently, the samples were dehydrated and cleared. Joints were embedded in paraffin, and 6 μm sections were taken through the entire joint at 80-μm intervals. Slides were stained with safranin-O and fast green. Each knee produced 10–12 slides for scoring by three blinded observers. Histological scoring based on the OARSI grading system (grades 0–6)^55^ was performed on the medial tibial plateau. The results are expressed as the mean ± 95% confidence interval of the maximum score. Immunohistochemistry was performed to assess Col II and aggrecan levels. Inflammation of the synovial membrane is directly linked to clinical symptoms, such as joint swelling, synovitis and inflammatory pain^43^. The synovial membranes were stained with H&E to observe the appearance of synovitis. Additionally, joint sections were used for immunofluorescence staining of iNOS (inflammation marker). ROS production in vivo was determined using dihydroethidium following previously described protocols with modifications^56^. In brief, 24 h before euthanasia, each mouse received a 200 μl intravenous injection of dihydroethidium at 25 mg kg^−1^.

### Behavioural testing

Osteoarthritis-associated pain was measured using the von Frey assay and the hot-plate assay^57^. The two tests were performed three times before ACLT surgery and once every 2 weeks after surgery. To measure the response latencies in the hot-plate assay, a glass cylinder was used to keep mice on the hot surface of the plate, which was maintained at a temperature of 55 ± 0.5 °C. The time (in seconds) between placement of the mouse and the onset of paw shaking, licking or jumping behaviour was recorded as the index of response latency. The development of mechanical allodynia was assessed using an electronic von Frey anaesthesiometer. The withdrawal threshold was defined as the force (*g*) sufficient to elicit the withdrawal response. Mouse gait was analysed using an automated gait-analysis system (MGT-PR, Zhenghua Equipment) to assess motor performance. A video camera recorded from below while each mouse walked unforced across an illuminated gate platform. The software performed statistical analysis on the basis of the footprints and body-weight distribution. We performed automated gait analysis before surgery and 8 and 12 weeks after surgery.

### In vivo systemic toxicity experiments

After the mice were killed, the main organs (heart, liver, kidney, lung and spleen) were collected for H&E staining to evaluate systematic pathological changes.

### Statistical analysis

Statistical comparisons of two independent groups were performed using unpaired two-tailed *t*-test. Multiple comparisons were performed using one-way analysis of variance (ANOVA) with post-hoc Tukey test. Data based on ordinal grading systems were analysed using nonparametric Kruskal–Wallis test followed by Dunn post-hoc test. Each *n* indicates the number of biologically independent samples, whether mice per group or human specimens. Statistical analysis was performed using Excel and GraphPad Prism v.9.0. Statistical tests were processed using GraphPad Prism v.9.0 unless otherwise specified, and exact *P* values are provided in the figures whenever available (when *P* values are smaller than 0.0001, *P* < 0.0001 is shown, as the exact *P* value is not available in GraphPad Prism). Significance was set at *P* < 0.05, and the error bars represent the standard deviation for parametric data and the calculated 95% confidence intervals for nonparametric data. Data in Figs. 1c–e,g,h,m, 2a,b,h,i,l,m and 3a–f and Extended Data Figs. 1b–h, 3e, 4a–d,f,g, 5d–l, 8a and 10 were successfully replicated in two independent experiments.

### Reporting summary

Further information on research design is available in the Nature Portfolio Reporting Summary linked to this article.

## Online content

Any methods, additional references, Nature Portfolio reporting summaries, source data, extended data, supplementary information, acknowledgements, peer review information; details of author contributions and competing interests; and statements of data and code availability are available at 10.1038/s41586-022-05499-y.

## Supplementary information

Supplementary Information Supplementary text regarding tissue penetration of CM-NTUs and how CM-NTUs improve cell anabolism.

Reporting Summary

Supplementary Fig. 1 Uncropped gels presented in the paper.

Supplementary Table 1 Proteomics results of NTUs.

Supplementary Table 2 NTU-specific peptides corresponding to photosynthetic activity-related proteins.

Supplementary Table 3 Proteomics results of CM.

Supplementary Table 4 All detected metabolites.

Supplementary Table 5 List of qPCR primers used in this study.

## Data Availability

All relevant data are available from the corresponding authors upon reasonable request. The transcriptomics data are available at NCBI BioProject under accession number PRJNA744581. Our metabolomics data were analysed using the Reactome database (https://www.reactome.org/). Source data are provided with this paper.

## Source data

Source Data Fig. 1
Source Data Fig. 2
Source Data Fig. 3
Source Data Fig. 5
Source Data Extended Data Fig. 1
Source Data Extended Data Fig. 3
Source Data Extended Data Fig. 4
Source Data Extended Data Fig. 5
Source Data Extended Data Fig. 8
Source Data Extended Data Fig. 9
