# Supplementary material for: A plant-derived natural photosynthetic system for improving cell anabolism
Source: Nature. 2022 Dec 7;612(7940):546–54. doi: 10.1038/s41586-022-05499-y (PMC9750875; doi:10.1038/s41586-022-05499-y)
Supplement: Supplementary file 1 — Supplementary text regarding tissue penetration of CM-NTUs and how CM-NTUs improve cell anabolism. [file 41586_2022_5499_MOESM1_ESM.pdf]

nature portfolio

https://doi.org/10.1038/s41586-022-05499-y

Supplementary information

A plant-derived natural photosynthetic system for improving cell anabolism

In the format provided by the authors and unedited

Supplementary Text

Superior tissue penetration of the CM-NTUs

By detecting the fluorescence intensity in the chondrocytes and residual solution after coculture, we found the fluorescence intensity reached the highest value after 1 hour of CM-NTU stimulation, gradually decreased after replacing the fresh culture medium, and reached a plateau after 8 hours (Fig. 2l). The number of NTUs per cell estimated by fluorescence intensity decreased from ~3.8×10^4 to ~2.5×10^4 in the CM-NTU group in the first 8 hours. However, pretreatment with GW4869 clearly slowed the decrease in fluorescence intensity in chondrocytes (the number of NTUs per cell decreased from ~3.8×10^4 to ~3.3×10^4 in 8 hours; Fig. 2l). Furthermore, the fluorescence signal in the non-fluorescently labeled medium could appear during the process of culturing, and the CM-NTU group had a significantly higher fluorescence signal (~1.4×10^4 NTUs per cell was released into the supernatant) than the CM-NTU + GW4869 group (~3.8×10^3 NTUs released per cell) during the first 8 hours of culturing (Fig. 2m).

The CM-NTUs improve cell anabolism

We compared the differences in ATP production by NTUs *in vitro* (cell-free) and *in vivo* (cell), and the results showed that NTUs increased the cellular ATP concentration at a rate of 5.2×10^5 ATP s^{-1} per cell, which is slower than the theoretical value of ATP that NTUs can produce in cells (calculated according to the number of NTUs in a single cell and the rate at which NTUs produce ATP *in vitro*). We speculate that the difference between the actual value and the theoretical value is because of the difference between reaction systems (such as substrate and cofactor concentrations).
