# Supplementary material for: A plant-derived natural photosynthetic system for improving cell anabolism
Source: Nature. 2022 Dec 7;612(7940):546–54. doi: 10.1038/s41586-022-05499-y (PMC9750875; doi:10.1038/s41586-022-05499-y)
Supplement: Supplementary file 2 — Reporting Summary [file 41586_2022_5499_MOESM2_ESM.pdf]

nature portfolio

Corresponding author(s): Xian-Feng Lin

Last updated by author(s): 2022-08-21

Reporting Summary

Nature Portfolio wishes to improve the reproducibility of the work that we publish. This form provides structure for consistency and transparency in reporting. For further information on Nature Portfolio policies, see our Editorial Policies and the Editorial Policy Checklist.

Statistics

For all statistical analyses, confirm that the following items are present in the figure legend, table legend, main text, or Methods section.

n/a	Confirmed	
☐	☒	The exact sample size (n) for each experimental group/condition, given as a discrete number and unit of measurement
☐	☒	A statement on whether measurements were taken from distinct samples or whether the same sample was measured repeatedly
☐	☒	The statistical test(s) used AND whether they are one- or two-sided<br>*Only common tests should be described solely by name; describe more complex techniques in the Methods section.*
☐	☒	A description of all covariates tested
☐	☒	A description of any assumptions or corrections, such as tests of normality and adjustment for multiple comparisons
☐	☒	A full description of the statistical parameters including central tendency (e.g. means) or other basic estimates (e.g. regression coefficient) AND variation (e.g. standard deviation) or associated estimates of uncertainty (e.g. confidence intervals)
☐	☒	For null hypothesis testing, the test statistic (e.g. F, t, r) with confidence intervals, effect sizes, degrees of freedom and P value noted<br>*Give P values as exact values whenever suitable.*
☒	☐	For Bayesian analysis, information on the choice of priors and Markov chain Monte Carlo settings
☒	☐	For hierarchical and complex designs, identification of the appropriate level for tests and full reporting of outcomes
☐	☒	Estimates of effect sizes (e.g. Cohen's d, Pearson's r), indicating how they were calculated

Our web collection on statistics for biologists contains articles on many of the points above.

Software and code

Policy information about availability of computer code

Data collection	No software was used.
Data analysis	All statistical analyses were performed on Excel and GraphPad Prism v.9.0. Image J (Version 1.48h3) was used for fluorescence-image analysis. Transcriptomics data were analyzed online with a Cloud Platform (http://www.genome.cn/ann_cloud). FlowJo (v.10) was used for flow cytometry analysis. The reconstructed images of µCT were analyzed using Version 3.1 software (SKYSCAN 1275).

For manuscripts utilizing custom algorithms or software that are central to the research but not yet described in published literature, software must be made available to editors and reviewers. We strongly encourage code deposition in a community repository (e.g. GitHub). See the Nature Portfolio guidelines for submitting code & software for further information.

Data

Policy information about availability of data

All manuscripts must include a data availability statement. This statement should provide the following information, where applicable:

- Accession codes, unique identifiers, or web links for publicly available datasets
- A description of any restrictions on data availability
- For clinical datasets or third party data, please ensure that the statement adheres to our policy

The source data underlying Figs. 1, 2, 3, and 5, and Extended Data Figs. 1, 3, 4, 5, 8, and 9 are provided as a Source Data file. All the relevant data are available from the authors upon reasonable request. The transcriptomic data are available at NCBI under Project PRJNA744581. Our metabolomics data were analyzed with the Reactome database (https://www.reactome.org/). A reporting summary for this article is available as a Supplementary Information file.

Human research participants

Policy information about studies involving human research participants and Sex and Gender in Research.

Reporting on sex and gender	The study lacks sex- and gender-based analysis. As a proof of concept, the focus of this paper is the development of a plant-derived natural photosynthetic system for improving cell anabolism.
Population characteristics	Human degenerated cartilage (n=4, mean age±standard deviation= 67 ±10, 1 male, 3 females) was obtained from OA patients undergoing total knee replacement. Human healthy cartilage (n=5, mean age±standard deviation= 47 ±14, 4 males, 1 female) was obtained from patients with amputations after severe lower extremity injuries (with no history of OA).
Recruitment	Tissue specimens were obtained from discarded tissue from patients, per "Population characteristics" above. No selection biases were present.
Ethics oversight	The study design and protocol were approved by the Ethics Committee of Sir Run Run Shaw Hospital. Written informed consent was obtained from every patient.

Note that full information on the approval of the study protocol must also be provided in the manuscript.

Field-specific reporting

Please select the one below that is the best fit for your research. If you are not sure, read the appropriate sections before making your selection.

☒ Life sciences ☐ Behavioural & social sciences ☐ Ecological, evolutionary & environmental sciences

For a reference copy of the document with all sections, see nature.com/documents/nr-reporting-summary-flat.pdf

Life sciences study design

All studies must disclose on these points even when the disclosure is negative.

Sample size	Sample sizes of in vitro experiments were determined according to previous experimental experience. The sample sizes of in vitro experiments refers to previously published literature (Nature Nanotechnology, 2018, 13, 1182–1190). For in vivo experiments, we performed prospective power analysis by using G*Power analysis. Effect sizes were estimated on the basis of previous experiments in our group and in the published literature (Wang, Ann Rheum Dis, (2020), 79; Li, Sci Transl Med, (2019), 11). The probability values of type I and type II error were set at 0.05 and 0.20, respectively. Power analysis showed that at least 8 mice in each group were needed. We increased the number of mice to 12 per group. In the mouse cohorts (12-week-old male mice) used for ATP and NADPH analysis, tissues from the whole femoral and tibial articular surfaces were isolated and identified, with n = 10 per group based on power analysis using preliminary data.
Data exclusions	No data were excluded.
Replication	All experimental data are given including replicates. Details of experimental replicates are given in the figure legends. All reported attempts at replication were successful.
Randomization	All samples/organisms were randomly allocated into experimental groups.
Blinding	Blinding and randomization were applied to all experiments.

Reporting for specific materials, systems and methods

We require information from authors about some types of materials, experimental systems and methods used in many studies. Here, indicate whether each material, system or method listed is relevant to your study. If you are not sure if a list item applies to your research, read the appropriate section before selecting a response.

Materials & experimental systems

n/a	Involved in the study	
☐	☒	Antibodies
☐	☒	Eukaryotic cell lines
☒	☐	Palaeontology and archaeology
☐	☒	Animals and other organisms
☒	☐	Clinical data
☒	☐	Dual use research of concern

Methods

n/a	Involved in the study	
☒	☐	ChIP-seq
☐	☒	Flow cytometry
☒	☐	MRI-based neuroimaging

Antibodies

Antibodies used

Anti-beta I Tubulin antibody [EPR16778] (Abcam, Cat. No. ab179511)
Anti-Sodium Potassium ATPase antibody [EP1845Y] (Abcam, Cat. No. ab76020)
Collagen Type II Polyclonal antibody (Proteintech, Cat No. 28459-1-AP)
Aggrecan Polyclonal Antibody (Proteintech, Cat No. 13880-1-AP)
MMP13 Polyclonal Antibody (Proteintech, Cat No. 18165-1-AP)
Anti-ADAMTS5 antibody (Abcam, Cat. No. ab246975)
SIRT1 Polyclonal antibody (Proteintech, Cat No. 13161-1-AP)
PGC1a Monoclonal antibody (Proteintech, clone 1C1B2, Cat No. 66369-1-Ig)
TFAM Polyclonal antibody (Proteintech, Cat No. 22586-1-AP)
NRF1 Polyclonal antibody (Proteintech, Cat No. 12482-1-AP)
NRF2 Polyclonal antibody (Proteintech, Cat No. 16396-1-AP)
Anti-iNOS antibody [EPR16635] (Abcam, Cat. No. ab178945)
MYOD1 Polyclonal antibody (Proteintech, Cat No. 18943-1-AP)
Anti-Myogenin antibody [EPR4789] (Abcam, Cat. No. ab124800)
Anti-PsbD (D2) antibody (Agrisera, Cat. No.AS06146)
Anti-PsbA (D1) antibody (Agrisera, Cat. No.AS05084)
Anti-AtpB antibody (Agrisera, Cat. No.AS05085)
Beta Actin Polyclonal antibody (Proteintech, Cat No. 20536-1-AP)
Anti-rabbit IgG HRP-linked secondary antibody (FDbio science, Cat No. FDR007, 1:5,000)
Anti-mouse IgG HRP-linked secondary antibody (FDbio science, Cat No. FDM007, 1:5,000)
CoraLite488-conjugated goat anti-rabbit IgG (Proteintech, Cat No. SA00013-2, 1:500)
Goat anti-rabbit IgG secondary antibody (Thermo Fisher, Cat No. 31460, 1:500)

Validation

All antibodies were validated by the commercial supplier. All validation statements can be found on the respective antibody website:
Anti-beta I Tubulin antibody [EPR16778]: https://www.abcam.cn/beta-i-tubulin-antibody-epr16778-ab179511.html
Anti-Sodium Potassium ATPase antibody [EP1845Y]: https://www.abcam.cn/sodium-potassium-atpase-antibody-ep1845y-plasma-membrane-loading-control-ab76020.html
Collagen Type II Polyclonal antibody: http://www.ptgcn.com/products/Collagen-Type-II-Antibody-28459-1-AP.htm
Aggrecan Polyclonal Antibody: http://www.ptgcn.com/products/ACAN-Antibody-13880-1-AP.htm
MMP13 Polyclonal Antibody: http://www.ptgcn.com/products/MMP13-Antibody-18165-1-AP.htm
Anti-ADAMTS5 antibody: https://www.abcam.cn/adamts5-antibody-ab246975.html
SIRT1 Polyclonal antibody: https://www.ptgcn.com/products/SIRT1-Antibody-13161-1-AP.htm
PGC1a Monoclonal antibody [1C1B2]: https://www.ptgcn.com/products/PPARGC1A-Antibody-66369-1-Ig.htm
TFAM Polyclonal antibody: https://www.ptgcn.com/products/TFAM-Antibody-22586-1-AP.htm
NRF1 Polyclonal antibody: https://www.ptgcn.com/products/NRF1-Antibody-12482-1-AP.htm
NRF2 Polyclonal antibody: https://www.ptgcn.com/products/NFE2L2,NRF2-Antibody-16396-1-AP.htm
Anti-iNOS antibody [EPR16635]: https://www.abcam.cn/inos-antibody-epr16635-ab178945.html
MYOD1 Polyclonal antibody: http://www.ptgcn.com/products/MYOD1-Antibody-18943-1-AP.htm
Anti-Myogenin antibody [EPR4789]: https://www.abcam.cn/myogenin-antibody-epr4789-ab124800.html
Anti-PsbD (D2) antibody: https://www.agrisera.com/en/artiklar/psbd-d2-global-antibody.html
Anti-PsbA (D1) antibody: https://www.agrisera.com/en/artiklar/psba-d1-protein-of-psii-c-terminal-100-l.html
Anti-AtpB antibody: https://www.agrisera.com/en/artiklar/atpb-beta-subunits-of-atp-synthase-global-antibody.html
Beta Actin Polyclonal antibody: https://www.ptgcn.com/products/ACTB-Antibody-20536-1-AP.htm
Anti-rabbit IgG HRP-linked secondary antibody: http://www.fdbio.net/productinfo.php?id=219
Anti-mouse IgG HRP-linked secondary antibody: http://www.fdbio.net/productinfo.php?id=220
CoraLite488-conjugated goat anti-rabbit IgG: https://www.ptgcn.com/products/CoraLite488-conjugated-Affinipure-Goat-Anti-Rabbit-IgG-H-L-secondary-antibody.htm
Goat anti-rabbit IgG secondary antibody: https://www.thermofisher.cn/cn/zh/antibody/product/Goat-anti-Rabbit-IgG-H-L-Secondary-Antibody-Polyclonal/31460

Eukaryotic cell lines

Policy information about cell lines and Sex and Gender in Research

Cell line source(s)

RAW 264.7 murine macrophages, a human umbilical vein endothelial cell line (HUVEC), and a mouse fibroblast cell line (NIH/3T3) were obtained from the China Center for Type Culture Collection (CCTCC).

Authentication

The cell lines were not authenticated.

Mycoplasma contamination

All cell lines were tested for mycoplasma contamination. No mycoplasma contamination was found.

Commonly misidentified lines
(See ICLAC register)

No commonly misidentified cell lines were used.

Animals and other research organisms

Policy information about studies involving animals; ARRIVE guidelines recommended for reporting animal research, and Sex and Gender in Research

Laboratory animals

Male C57BL/6 mice (4 weeks, 8 weeks, 12 weeks or 12 months), female C57BL/6 mice (12 weeks), and male Sprague-Dawley rats (4 [illegible]

weeks) were used. Animals were housed in groups of 4-6 mice per individually ventilated cage in a 12 h light dark cycle (06:30-18:30 light; 18:30-06:30 dark), with constant room temperature (21 ± 1 °C) and relative humidity (40-60 %). Animals had access to food and water ad libitum.

Wild animals

The study did not involve wild animals studies.

Reporting on sex

We induced experimental OA by ACLT surgery in 12-week-old male, 12-week-old female, and 12-month-old male mice, and the treatment experiment was repeated in these animal groups.

Field-collected samples

The study did not involve field-collected samples.

Ethics oversight

All animal studies were performed according to ethical regulations and protocols approved by the Sir Run Run Shaw Hospital Committee for Animal Resources and the Institutional Animal Care and Use Committee of Zhejiang Center of Laboratory Animals. All mouse experimental procedures were carried out following the Regulations for the Administration of Affairs Concerning Experimental Animals approved by the State Council of People's Republic of China.

Note that full information on the approval of the study protocol must also be provided in the manuscript.

Flow Cytometry

Plots

Confirm that:

☒ The axis labels state the marker and fluorochrome used (e.g. CD4-FITC).

☒ The axis scales are clearly visible. Include numbers along axes only for bottom left plot of group (a 'group' is an analysis of identical markers).

☒ All plots are contour plots with outliers or pseudocolor plots.

☒ A numerical value for number of cells or percentage (with statistics) is provided.

Methodology

Sample preparation

Effects of endocytosis inhibitors on the cellular uptake of NPs
Sufficient chondrocytes were seeded in 12-well plates to reach 60–70% confluency after overnight incubation. The medium was replaced with fresh medium, and four endocytosis inhibitors (chlorpromazine, filipin III, wortmannin or cytochalasin D) were subsequently separately added to the medium at concentrations of 50, 7.5, 5 or 5 µM. After 30 min of preincubation, the cells were treated with DiI-labeled CM-NTUs (2 × 10^5 NTUs per cell) in the presence of the inhibitors for another 6 hours. Finally, the cells were trypsinized, isolated by centrifugation and resuspended in PBS. The fluorescence intensity in each well was quantitatively determined by flow cytometry (FACSCalibur).
Selectivity of chondrocytes taking up CM-NTUs
Equal amounts (1 × 10^5 cells) of chondrocytes (Hoechst 33342-labeled nuclei and DiI-labeled cell membranes), NPCs (Hoechst 33342-labeled nuclei and DiD-labeled cell membranes), SCs (DiI-labeled cell membranes), macrophages (DiD-labeled cell membranes), and fibroblasts (Hoechst 33342-labeled cell nuclei) were cocultured on Petri dishes and incubated overnight. CM-NTUs (DiO-labeled NTUs) at a concentration of 2 × 10^5 NTUs per cell were added and coincubated with these cells for 6 hours, and then flow cytometry was performed. In another competitive experiment, five kinds of cell membrane-coated NTUs in equal amounts (2 × 10^5 NTUs per cell) were coincubated with 2 × 10^5 chondrocytes. Due to the limited types of staining labels, two staining schemes were used in two parallel experiments. In the first experiment, chondrocyte nuclei were labeled with Hoechst 33342. The five different membrane-coated NTUs and their staining schemes were chondrocyte membrane-NTUs (CM-NTUs, NTUs labeled by DiO), nucleus pulposus cell membrane-NTUs (NPCM-NTUs, NTUs labeled by DiI), macrophage membrane-NTUs (MM-NTUs, NTUs labeled by DiD), muscle satellite cell membrane-NTUs (SCM-NTUs, unlabeled), and fibroblast membrane-NTUs (FM-NTUs, unlabeled). These five materials were added to the culture medium and cocultured with chondrocytes for 6 hours (Scheme #1). In the second experiment, NPCM-NTUs and MM-NTUs were not labeled, while SCM-NTUs and FM-NTUs were labeled with DiI and DiD, and the rest remained unchanged (Scheme #2). Then, flow cytometric analysis (LSRFortessa) was performed.
To clarify whether NTUs cause a cellular stress response, the production of reactive oxygen species (ROS) in cells containing NTUs under different red light illumination (8.9–320 µmol photons m^{-2} s^{-1}) was tested by flow cytometry (FACSCalibur) with a membrane-permeable fluorescent probe, DCFH-DA (Beyotime).

Instrument

BD FACSCalibur and BD LSRFortessa

Software

FlowJo(v.10)

Cell population abundance

No post-sort fractions were collected through the Flow cytometry.

Gating strategy

Cells of multi-fluorescence flow cytometry were identified with FSC-A/SSC-A gating and followed by FSC-H/FSC-A for singlets on BD LSRFortessa. Single cells for mono-fluorescence flow cytometry were identified with FSC/SSC gating on BD FACSCalibur. The boundaries between "positive" and "negative" were determined by the clear cell subpopulations and unstained negative controls. The gating strategy was detailly exhibited in Extended Data Figure 1i, Extended Data Figure 2.

☒ Tick this box to confirm that a figure exemplifying the gating strategy is provided in the Supplementary Information.
