# Supplementary figures and images for: A plant-derived natural photosynthetic system for improving cell anabolism
Source: Nature. 2022 Dec 7;612(7940):546–54. doi: 10.1038/s41586-022-05499-y (PMC9750875; doi:10.1038/s41586-022-05499-y)

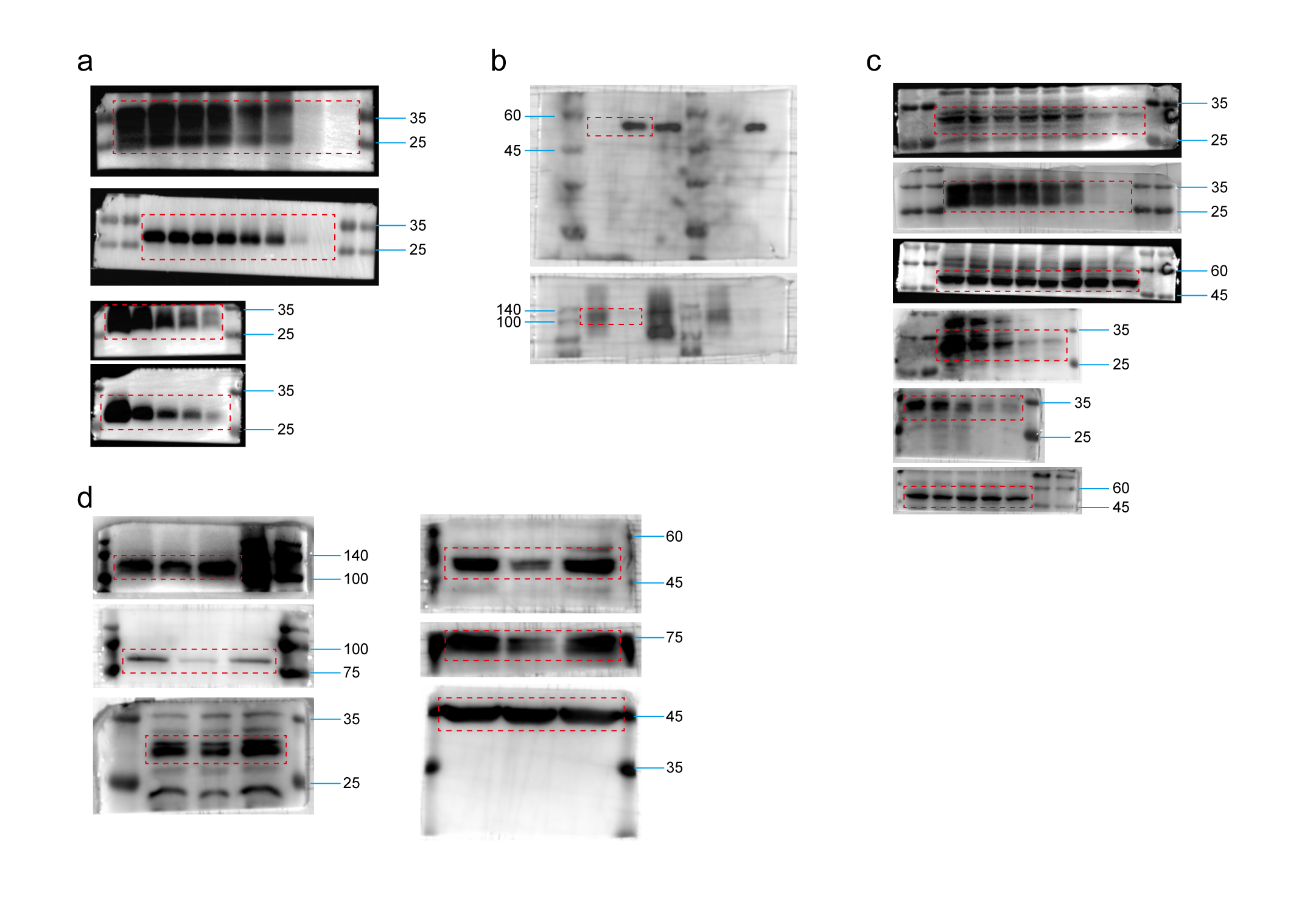

Supplement: Supplementary file 3 — Uncropped gels presented in the paper. [file 41586_2022_5499_MOESM3_ESM.tif]
